# Antidepressant-like and Anticonvulsant Effects of a Phenolic-Enriched Fraction from *Malpighia mexicana* in Murine Models

**DOI:** 10.3390/neurosci7040088

**Published:** 2026-08-10

**Authors:** David Osvaldo Salinas-Sánchez, Manasés González-Cortazar, Franceli Itzel Batalla-Martinez, Alejandro Zamilpa, Maura Téllez-Téllez, María Guadalupe Valladares-Cisneros, César Sotelo-Leyva, Rodolfo Figueroa-Brito, Ma Dolores Pérez-García, Dante Avilés-Montes

**Affiliations:** 1 Centro de Investigación en Biodiversidad y Conservación, Universidad Autónoma del Estado de Morelos, Av. Universidad No. 1001, Col. Chamilpa, Cuernavaca 62209, Morelos, Mexico; davidos@uaem.mx; 2 Escuela de Estudios Superiores del Jicarero, Universidad Autónoma del Estado de Morelos, Galeana-Tequesquitengo S/N, Col. El Jicarero, Jojutla 62909, Morelos, Mexico; 3 Centro de Investigación Biomédica del Sur, Instituto Mexicano del Seguro Social, Argentina No. 1, Col. Centro, Xochitepec 62790, Morelos, Mexicolola_as@yahoo.com.mx (M.D.P.-G.); 4 Maestría en Manejo de Recursos Naturales, Centro de Investigaciones Biológicas, Universidad Autónoma del Estado de Morelos, Av. Universidad No. 1001, Col. Chamilpa, Cuernavaca 62209, Morelos, Mexico; franceli.batalla@uaem.edu.mx; 5 Centro de Investigaciones Biológicas, Universidad Autónoma del Estado de Morelos, Av. Universidad No. 1001, Col. Chamilpa, Cuernavaca 62209, Morelos, Mexico; maura.tellez@uaem.mx; 6 Facultad de Ciencias Químicas e Ingeniería, Universidad Autónoma del Estado de Morelos. Av. Universidad No. 1001, Col. Chamilpa, Cuernavaca 62209, Morelos, Mexico; mg.valladares@uaem.mx; 7 Facultad de Ciencias Químico-Biológicas, Universidad Autónoma de Guerrero, Av. Lázaro Cárdenas S/N, Ciudad Universitaria Sur, Chilpancingo 39000, Guerrero, Mexico; cesarsotelo@uagro.mx; 8 Centro de Desarrollo de Productos Bióticos, Instituto Politécnico Nacional, Carretera Yautepec-Jojutla Km 6, AP24, San Isidro, Yautepec 62731, Morelos, Mexico; rfigueroa@ipn.mx; 9 Facultad de Ciencias Biológicas, Universidad Autónoma del Estado de Morelos, Av. Universidad No. 1001, Col. Chamilpa, Cuernavaca 62209, Morelos, Mexico

**Keywords:** *Malpighia mexicana*, kaempferol, p-coumaric acid, antidepressant-like effect, anticonvulsant activity, pentylenetetrazole, forced swimming test, tail suspension test, phenolic compounds

## Abstract

*Malpighia mexicana* has been traditionally used in Mexican ethnomedicine for the treatment of central nervous systems disorders. Previous studies have demonstrated neuropharmacological activity of crude extracts, but the contribution of enriched phytochemical fractions remains unclear. A phenolic-enriched fraction (G4) obtained from the aerial parts of *M. mexicana* was evaluated for antidepressant-like and anticonvulsant activities in mice. Antidepressant-like effects were assessed using the forced swimming test (FST) and tail suspension test (TST), while locomotor activity was evaluated in the open field test. Anticonvulsant activity was determined using the pentylentetrazole (PTZ)-induced seizure model. The phytochemical profile of G4 was characterized by HPLC-DAD analysis. G4 (50 and 200 mg/kg, p.o.) significantly reduced immobility time in both FST and TST compared to the vehicle group (*p* < 0.05) without affecting locomotor activity in the OFT, suggesting a specific antidepressant-like effect. In the PTZ model, G4 significantly increased latency to clonic and tonic seizures and reduced seizure severity. HPLC-DAD analysis revealed the presence of phenolic compounds, with kaempferol and p-coumaric acid as major constituents. The phenolic-enriched G4 fraction from *M. mexicana* exhibits antidepressant-like and anticonvulsant effects in murine models, supporting its traditional use. These findings suggest that phenolic constituents, particularly kaempferol and p-coumaric acid, may contribute to the observed neuropharmacological activity. However, since only the G4 fraction was pharmacologically evaluated and not the isolated compounds, the specific role of each constituent remains to be established. Further studies are required to elucidate the underlying mechanisms of action and the contribution of individual compounds and potential synergistic interactions.

## 1. Introduction

Globally, mental and neurological disorders are a serious public health problem; among these, depression and epilepsy are among the most prevalent and disabling conditions [1,2]. Depression is one of the leading causes of disability and is estimated to affect 332 million people worldwide; epilepsy affects nearly 50 million people, 70% of whom live in low- and middle-income countries without access to adequate pharmacological treatment [3,4,5,6]. It is estimated that 3.6 million people in Mexico suffer from depression and 2 million from epilepsy [7,8]. Pharmacological treatment for these conditions includes antidepressants (e.g., selective serotonin reuptake inhibitors, selective serotonin-norepinephrine reuptake inhibitors) and anticonvulsants (e.g., benzodiazepines, sodium channel modulators). However, undesirable side effects have been reported due to their use, such as sedation, falls, cognitive impairment, gastrointestinal disorders, and risk of dependence. This limits patients’ adherence to their treatment and, consequently, their quality of life [9,10,11,12]. As a result, there is growing interest in identifying safer therapeutic alternatives derived from natural sources. The World Health Organization (WHO) estimates that 80% of the world’s population relies on traditional medicine to meet their primary care needs [13,14]. In Mexico, particularly in rural and indigenous communities, the use of medicinal plants and traditional medicine remains a fundamental component of healthcare [15,16].

In this case, the species *Malpighia mexicana* A. Juss. (Malpighiaceae), known locally as guachocote, is a plant with edible fruit that is native to Mexico and has been widely used in Mexican traditional medicine since pre-Hispanic times [17,18,19]. Our research group previously demonstrated that an acetone extract of the leaves exhibits antidepressant, anticonvulsant, and anxiolytic activity in mice [20]. Neuropharmacological properties have been demonstrated in other species of the Malpighiaceae family. For example, *Byrsonima crassifolia* and *Heteropterys brachiata* have shown antidepressant and anticonvulsant effects in animal models, attributed to their high content of phenolic compounds, including flavonoids [21,22]. Studies conducted on related species identified kaempferol and p-coumaric acid as neuroactive phenolic compounds. Kaempferol, a flavonol, has anti-inflammatory effects, reduces oxidative stress, and modulates monoaminergic systems, thereby exhibiting antidepressant effects in the forced swimming test (FST) and tail suspension test (TST) models. It also exhibited an anticonvulsant effect in vivo studies using the PTZ-induced seizure model (PTZt) by reducing neuronal hyperexcitability. It also exhibits a neuroprotective effect against edema and necrosis and has a preventive effect against calcium-induced toxicity [23,24,25,26].

On the other hand, it has been reported that p-coumaric acid protects neuronal cells because it has antioxidant and anti-inflammatory properties. It reduces oxidative stress and exerts a neuroprotective effect by inhibiting apoptosis and improving mitochondrial function [27]. It has also been reported to have antidepressant effects and to improve memory by inhibiting neuroinflammation mediated by multiple targets and signaling pathways, such as AGE-RAGE, PKA/CREB/BDNF, and TLR [28,29]. However, the presence and pharmacological contribution of these compounds in *M. mexicana* remain unclear.

We hypothesize that a fraction enriched in phenolic compounds (G4) from *M. mexicana* exerts antidepressant-like and anticonvulsant effects and that these effects may be mediated, at least in part, by its major constituents kaempferol and p-coumaric acid, based on their known neuropharmacological properties reported in the literature. However, direct experimental confirmation of this hypothesis requires future studies with the isolated compounds. The objectives of this study were, therefore, (1) to perform the phytochemical characterization of the bioactive fraction G4 isolated from the acetone extract of *M. mexicana* leaves; (2) to evaluate its antidepressant and anticonvulsant effects in murine models; and (3) to identify the major phenolic constituents of the bioactive fraction G4.

## 2. Materials and Methods

### 2.1. Collection and Identification of the Species

Leaves of *M. mexicana* A. Juss. were collected in the southern region of the state of Morelos, in the community of Chiconcuac, municipality of Xochitepec, during September 2024 (18°47′3.9766″ N, 99°11′42.193″ W; 1171 m.a.s.l.). The taxonomic identification of the species was confirmed by M.C. Gabriel Flores, a specialist in plant taxonomy. A reference specimen was deposited in the HUMO Herbarium of the Center for Biodiversity and Conservation Research at the Autonomous University of the State of Morelos, with catalog number 35164.

### 2.2. Preparation of the Acetone Extract (MmAE)

The dried, powdered plant material (1.384 kg) was extracted with acetone (8 L) by maceration, undergoing three consecutive cycles of 72 h each, as described previously [20]. The acetone solvent was completely removed by reduced-pressure distillation using a Büchi R-215 rotary evaporator (EQUIPAR, Mexico City, Mexico), followed by further drying under high vacuum. The resulting extract (MmAE) was stored at 4 °C until chromatographic fractionation.

### 2.3. MmAE Chromatographic Fractionation

The MmAE extract (71 g) was fractionated by column chromatography using 100 g of silica gel 60 as the stationary phase. Elution was performed using a gradient system with *n*-hexane and ethyl acetate as the mobile phase [20], starting with 100% of the less polar solvent; gradients of *n*-hexane-ethyl acetate (95:5, 90:10, 85:15, 80:20, 75:25, 70:30, 65:35, 60:40, 55:45, 50:50, 30:70, 10:90 *v*/*v*), followed by elution with 100% of the more polar solvent. Finally, the column was washed with 100% methanol. A total of 29 fractions of 250 mL each were collected. The solvents were completely removed by reduced-pressure distillation using a Büchi R-215 rotary evaporator (EQUIPAR, CDMX, Mexico). The resulting fractions were analyzed by thin-layer chromatography (TLC) and grouped according to similar chromatographic profiles, resulting in 6 groups (G1 to G6). Fraction G4, containing two major compounds, was evaluated. Based on our previous study [20], these six fractions were preliminarily screened for neuropharmacological activity. Among them, fraction G4 exhibited the most promising antidepressant-like and anticonvulsant effects in initial behavioral assays. Therefore, G4, which contained two major compounds, was selected for detailed phytochemical characterization and comprehensive pharmacological evaluation in the present study.

#### Isolation and Identification of Kaempferol and p-Coumaric Acid

G4 (1 g) was subjected to column chromatography using 10 g of RP-18 reverse-phase silica gel. Elution was performed using an H_2_O–acetonitrile gradient system, starting with 100% H_2_O and continuing with decreasing proportions of polarity down to a 30:70% H_2_O–acetonitrile mixture, followed by washes with 100% acetonitrile, methanol, and acetone. Sixteen 20 mL fractions were collected, which were concentrated under reduced pressure in a Büchi R-215 rotary evaporator. Fractions 9 through 13 exhibited a single spot on TLC and fluoresced with an intense blue color under long-wave UV (310 nm). The compound was identified by HPLC and UPLC-MS analysis as p-coumaric acid (**1**). Fraction 16 also exhibited a single spot on CCF with a phosphorescent green color under long-wave UV (365 nm), which was identified by HPLC, UPLC-MS, and NMR as kaempferol (**2**). The complete NMR spectra used for structural elucidation are provided in the Supplementary Materials (Figures S1–S6).

### 2.4. Analytical Methods

#### 2.4.1. HPLC-DAD Analytical Conditions

An HPLC-DAD analysis of the MmAE extract was performed to identify its chemical composition. The analysis was conducted using a Waters 2695 liquid chromatograph (Waters, Milford, MA, USA) equipped with a Waters 2996 photodiode array detector (Waters, Milford, MA, USA). Chromatographic separation was performed on a Superspher RP C18 column (120 × 4 mm, 5 µm; Merck, Darmstadt, Germany). The mobile phase consisted of solvent A (HPLC-grade water containing 0.5% formic acid) and solvent B (acetonitrile), using the following gradient program: 100:0 (0–1 min), 95:5 (2–3 min), 70:30 (4–20 min), 50:50 (21–22 min), 20:80 (24–25 min), 0:100 (26–27 min), and 100:0 (28–30 min). The flow rate was set at 0.9 mL/min. Detection was monitored in a wavelength range of 200–400 nm. The injection volume was 10 µL. As an analytical standard, p-coumaric acid (Sigma-Aldrich, St. Louis, MO, USA) was used, with a purity of ≥98%, and kaempferol (Sigma-Aldrich, St. Louis, MO, USA), with a reported purity of approximately ≥90%.

The MmAE extract and compounds **1** and **2** were prepared at a concentration of [1 mg/mL] in HPLC-grade methanol containing 0.05% formic acid, and an injection volume of 5 µL was used. Compounds **1** and **2** were quantified by HPLC-DAD with detection at 310 nm (**1**) and 360 nm (**2**). Standard solutions were prepared in a concentration range of [6.25, 12.5, 25, and 50 µg/mL] for p-coumaric acid and [6.25, 12.5, 25, 50, and 100 µg/mL] for kaempferol to construct the calibration curve. Linearity was evaluated by linear regression, yielding the equation y = 48,454x + 30,378 with a coefficient of determination R^2^ = 0.9988 for compound **1** and y = 22,774x – 62,442 with a coefficient of determination R^2^ = 0.9986 for compound **2**. Sample concentrations were calculated from this equation using the area under the curve (AUC).

The ^1^H and ^13^C NMR spectra of kaempferol were recorded on a Bruker Avance III HD spectrometer operating at 500 MHz (^1^H) and 125 MHz (^13^C) using acetone-d6 as the solvent and tetramethylsilane (TMS) as the internal reference. The ^1^H NMR spectrum of p-coumaric acid was acquired on a Spinsolve ULTRA 80 MHz benchtop NMR spectrometer (Magritek, Wellington, New Zealand) using methanol-d4 as the solvent. Mass spectrometric analyses were performed using a Waters triple quadrupole mass spectrometer (Waters Corporation, Milford, MA, USA) equipped with a ZSpray electrospray ionization (ESI) source (Waters Corporation, Milford, MA, USA) maintained at 150 °C. Argon was used as the collision gas at a flow rate of 0.10 mL min^−1^.

#### 2.4.2. Validation of the Analytical Method

The sensitivity of the method was evaluated by determining the detection limits (LOD) and quantification limits (LOQ) for the analyzed compounds **1** and **2**. The LOD and LOQ were estimated using the signal-to-noise ratio, calculated from the standard deviation of the blank (methanol) and the slope of the calibration curve, using the equations LOD = 3.3σ/S and LOQ = 10σ/S, where σ is the standard deviation of the response and S is the slope of the analytical curve [30].

### 2.5. Drugs

The drugs used were diazepam (DZP, catalog No. Y0000596), pentylenetetrazol (PTZ, catalog No. P6500), pentobarbital sodium (PBT, catalog No. Y0002194) and imipramine hydrochloride (IMI, catalog No. Y0000893), which were purchased from Sigma-Aldrich Chemical Co. (St. Louis, MO, USA).

### 2.6. Preparation of Administration Solutions

The G4 fraction was resuspended in 0.9% physiological saline with the addition of 0.5% Tween 80 as a dispersing agent. Doses of 50 and 200 mg/kg were prepared from this stock solution and administered orally. The vehicle used as a negative control consisted of 0.9% physiological saline with 0.5% Tween 80 (100 µL/10 g, p.o.). Imipramine hydrochloride (15 mg/kg, p.o.), diazepam (1 mg/kg, i.p.), and pentylenetetrazol (100 mg/kg, i.p.) were dissolved in sterile distilled water according to the manufacturer’s instructions. All solutions were freshly prepared on the day of the experiment.

### 2.7. Experimental Animals

Male CD-1 mice (5 weeks old, initial body weight approximately 25 g) were obtained from the Bioterio of the Faculty of Medicine at the Autonomous University of the State of Morelos (Cuernavaca, Morelos, Mexico). Animals were housed in groups of eight per cage under controlled environmental conditions (23 ± 1 °C, 50–60% relative humidity, and a 12 h light/dark cycle, lights on at 08:00 h), with free access to standard laboratory chow and water.

The present study was designed as an initial pharmacological screening using standardized acute behavioral models rather than as an investigation of age-dependent neurological mechanisms. Therefore, male CD-1 mice (5 weeks old) were selected for methodological reasons to ensure a homogeneous experimental population and to minimize biological variability. At this age, mice have completed the post-weaning period and exhibit stable locomotor, exploratory, and behavioral performance suitable for reproducible neuropharmacological testing. Furthermore, this age and body-weight range is consistent with that reported in numerous published studies employing CD-1 mice in behavioral models of depression and epilepsy, supporting the use of young adult animals in acute neuropharmacological screening. To minimize biological variability, all experimental groups consisted of age-matched animals from the same breeding facility, acclimatized under identical conditions and randomly assigned to treatment groups; moreover, for each neuropharmacological assay (FST, TST, OFT, and PTZ test), independent cohorts of mice (*n* = 8 per group) were used, with random assignment within each cohort and no reuse across tests, to avoid carryover effects or inter-test interference. The exclusive use of male animals was intended to reduce hormonal variability associated with the estrous cycle during this initial pharmacological evaluation, following current recommendations for rigorous experimental design and reporting [31,32,33,34,35,36].

Animals were allowed to acclimatize to the housing conditions for one week before the experiments. All behavioral tests were performed during the light phase between 09:00 and 16:00 h. Experimental procedures were conducted in accordance with the Mexican Official Standard NOM-062-ZOO-1999 [37] for the care and use of laboratory animals and complied with the ARRIVE 2.0 guidelines [38]. The experimental protocol was approved by the Institutional Research and Ethics Committee (approval number R-2023-1702-008).

### 2.8. Neuropharmacological Assays

#### 2.8.1. Forced Swimming Test (FST)

The forced swimming test (FST) is one of the most widely used behavioral assays for screening substances with antidepressant potential [32,39,40,41,42]. The test was conducted using Plexiglas cylinders (20 cm high × 12 cm in diameter) placed on a flat surface and filled with water (25 ± 1 °C) to a depth of 16 cm. The cylinders were separated by a wooden partition with six compartments, allowing for the simultaneous observation of six animals. Although a single 6 min session is frequently used in mice, the present study employed the original two-session forced swimming paradigm (15 min pre-exposure followed by a 5 min test session 24 h later). This protocol remains a validated approach for evaluating antidepressant-like activity when behavioral adaptation following prior swim exposure is of interest [39,43,44]. Moreover, this design was selected to maintain methodological consistency with our previous study on *Malpighia mexicana* [20], thereby allowing direct comparison between the crude extract and the phenolic-enriched fraction.

The experiment consisted of three phases. In the training phase, each mouse was placed individually in the water-filled cylinder for 15 min. After exposure, the animals were removed, gently dried with a clean cloth, and placed in cages lined with clean wood shavings, under an incandescent lamp to facilitate drying. In the second phase, twenty-four hours after the training session, mice received the assigned treatment, and one hour later they underwent the 5 min test session. The animals received the corresponding treatments: fraction G4 (50 or 200 mg/kg, p.o., *n* = 8), vehicle (0.9% saline solution with 0.5% Tween-80, 100 µL/10 g, p.o., *n* = 8) or imipramine (IMI, 15 mg/kg, p.o., positive control, *n* = 8), administered 1 h before the test. In the test phase, the animals were reintroduced into the cylinders, and their behavior was recorded for 5 min. The primary behavioral parameter used was immobility time, defined as the time during which the mice performed only the minimal movements necessary to stay afloat and breathe. All sessions were recorded with a video camera for subsequent analysis.

#### 2.8.2. Tail Suspension Test (TST)

The tail-suspension test has been established as a valid method for evaluating the activity of various antidepressant drugs and for screening drugs with antidepressant potential in a manner comparable to the forced swim test [45,46,47,48]. A 5 min observation period was selected because this duration has been widely validated for antidepressant screening in mice and was consistent with our previous studies [20].

The animals received the corresponding treatments: G4 fraction (50 or 200 mg/kg, p.o., *n* = 8), vehicle (0.9%, saline solution with 0.5% Tween-80, 100 µL/10 g, p.o., *n* = 8), or imipramine (IMI, 15 mg/kg, p.o., positive control, *n* = 8), administered 1 h before the test. The test was conducted in a custom-made black acrylic box (60 cm high × 60 cm wide × 60 cm deep); a metal bar was positioned horizontally at a height of 55 cm. The mice were individually suspended by the tail using adhesive tape placed 1 cm from the tip of the tail and centered on the horizontal bar. When the mice were suspended, the distance between their noses and the bottom of the apparatus was 30–35 cm. The duration of immobility was recorded for 5 min. Mice were considered immobile only when they hung passively and remained completely still. At the end of each session, the experimental arena was cleaned with a 10% ethanol solution to remove residues of urine and feces. The total duration of the test was monitored with a digital stopwatch, and all sessions were videotaped for subsequent analysis.

#### 2.8.3. Open Field Test (OFT)

This model is widely used to evaluate compounds with central nervous system activity, and in this case, it allows us to assess whether the treatments alter the physical and exploration abilities of the mice [49,50]. One hour before the experiment, the animals received the corresponding treatments: fraction G4 (50 or 200 mg/kg, p.o., *n* = 8), vehicle (0.9%, saline solution with 0.5% Tween-80, 100 µL/10 g, p.o., *n* = 8), or imipramine (IMI, 15 mg/kg, p.o., positive control, *n* = 8). The open-field test was conducted in a square acrylic box with a black bottom (30 × 30 × 15 cm), enclosed by transparent acrylic walls and divided into nine equal quadrants by white lines. Each mouse was placed individually in the center of the apparatus and allowed to explore freely for 5 min. The behavioral parameter recorded was the total number of crossings in the periphery and the center. At the end of each session, the experimental arena was cleaned with a 10% ethanol solution to remove residues of urine and feces. The total test duration was monitored with a digital stopwatch, and all sessions were videotaped for subsequent analysis.

#### 2.8.4. Pentylenetetrazol-Induced Seizures Test (PTZt)

PTZ-induced seizures reproduce several behavioral and electrographic features of generalized epileptic seizures observed in humans and therefore constitute one of the most widely used experimental models for evaluating anticonvulsant activity [51,52,53]. The mice received their respective treatments 1 h before the test: G4 (50 and 200 mg/kg, p.o., *n* = 8), vehicle (0.9%, saline solution with 0.5% Tween-80, 100 µL/10 g, p.o., *n* = 8), and diazepam (DZP, 1 mg/kg, i.p., positive control, *n* = 8). Subsequently, PTZ (100 mg/kg, i.p.) was administered to all animals, and they were individually observed for 30 min in transparent acrylic boxes to detect convulsive activity. The following parameters were recorded: latency of tonic and clonic seizures, number of clonic and tonic seizures, total number of seizures, and mouse survival.

### 2.9. Statistical Analysis

Data are presented as mean ± standard deviation. Normality and homogeneity of variances were assessed using the Shapiro–Wilk and Levene tests, respectively. To compare immobility times (forced swim and tail suspension) and seizure parameters (number of tonic, clonic, and generalized seizures and their respective latencies), a one-way analysis of variance (ANOVA) was used, followed by Tukey’s post hoc test (*p* < 0.05). For the survival variable, Kaplan–Meier curves were estimated and compared using the log-rank test. Statistical data processing and graph generation were performed using the AI-assisted analysis software Julius AI (Julius 1.1 Lite) (https://julius.ai) according to specifications defined by the authors. The results were verified by the authors.

## 3. Results

The dried and powdered leaves of *M. mexicana* (1.384 kg) were extracted with acetone by maceration, yielding 71 g of crude acetone extract (MmAE), which corresponded to a 5.1% yield on a dry weight basis.

### 3.1. Phytochemical Characterization by HPLC-DAD

#### HPLC of MmAE

HPLC-DAD analysis of the MmAE acetone extract and fraction G4 enabled the identification of phenolic compounds by comparing their retention times and UV absorption spectra with reference standards.

- Identification of p-coumaric acid (**1**)

**Figure 1:**
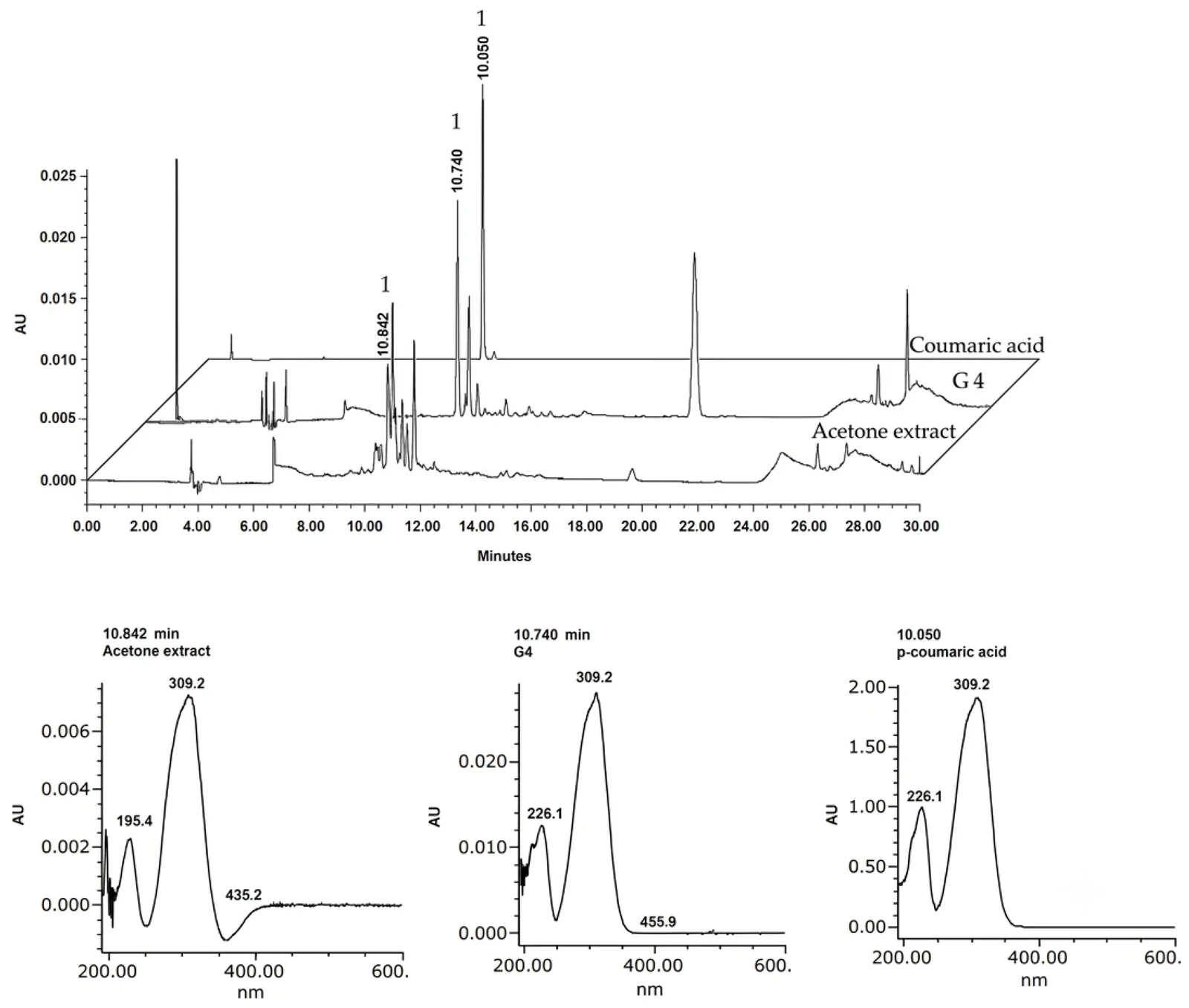
HPLC chromatograms (310 nm) of MmAE, G4, and the p-coumaric acid standard on a C18 column (acetonitrile/water gradient, 0.9 mL/min). The p-coumaric acid peak is indicated by (**1**). The UV spectrum (inset) shows maxima at 226 and 309 nm. Rt: p-coumaric acid = 10.05 min (standard), 10.74 min (G4), 10.84 min (MmAE).

**Figure 2:**
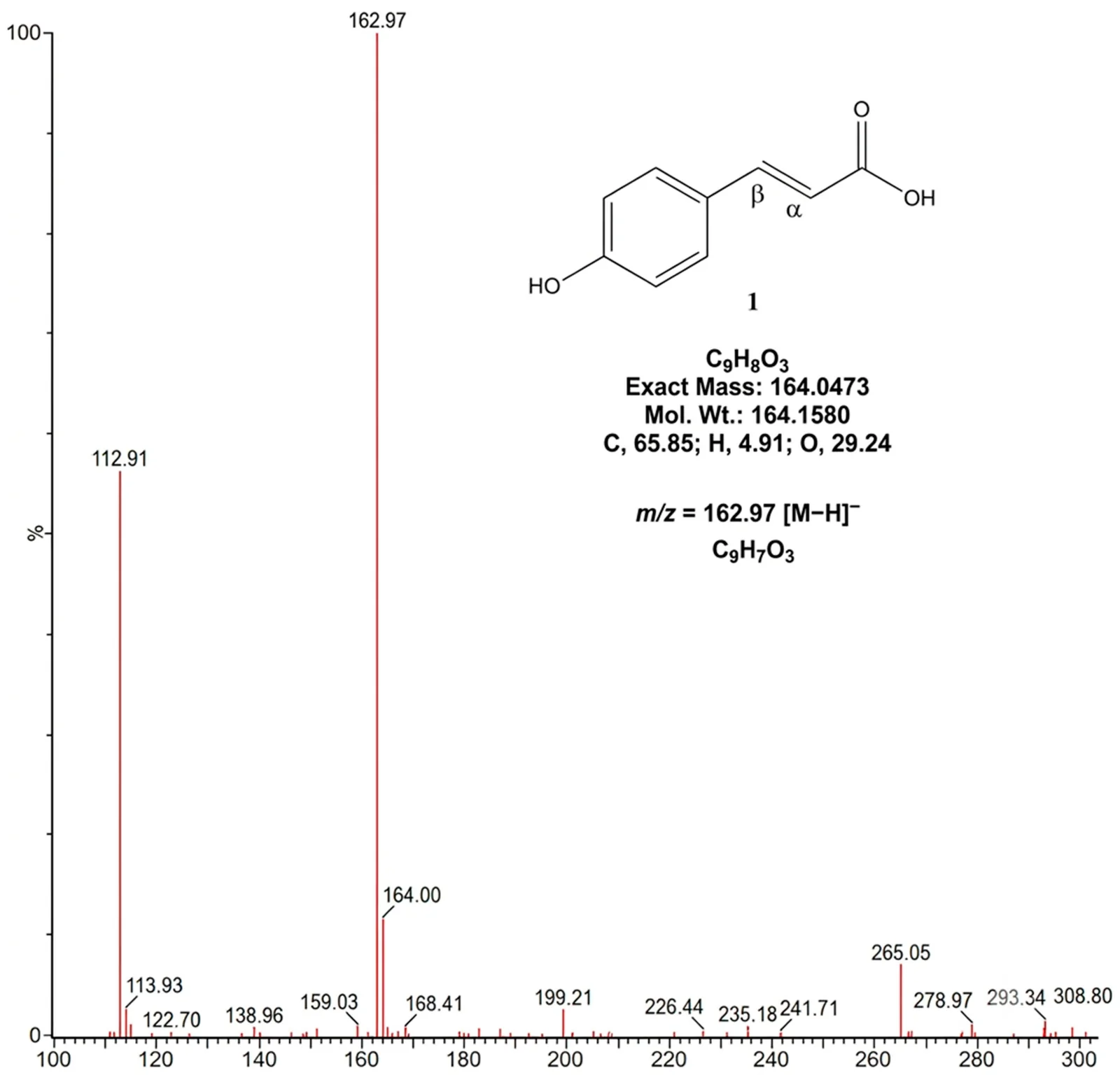
Mass spectrum (ESI-MS) of p-coumaric acid (**1**).

**Figure 3:**
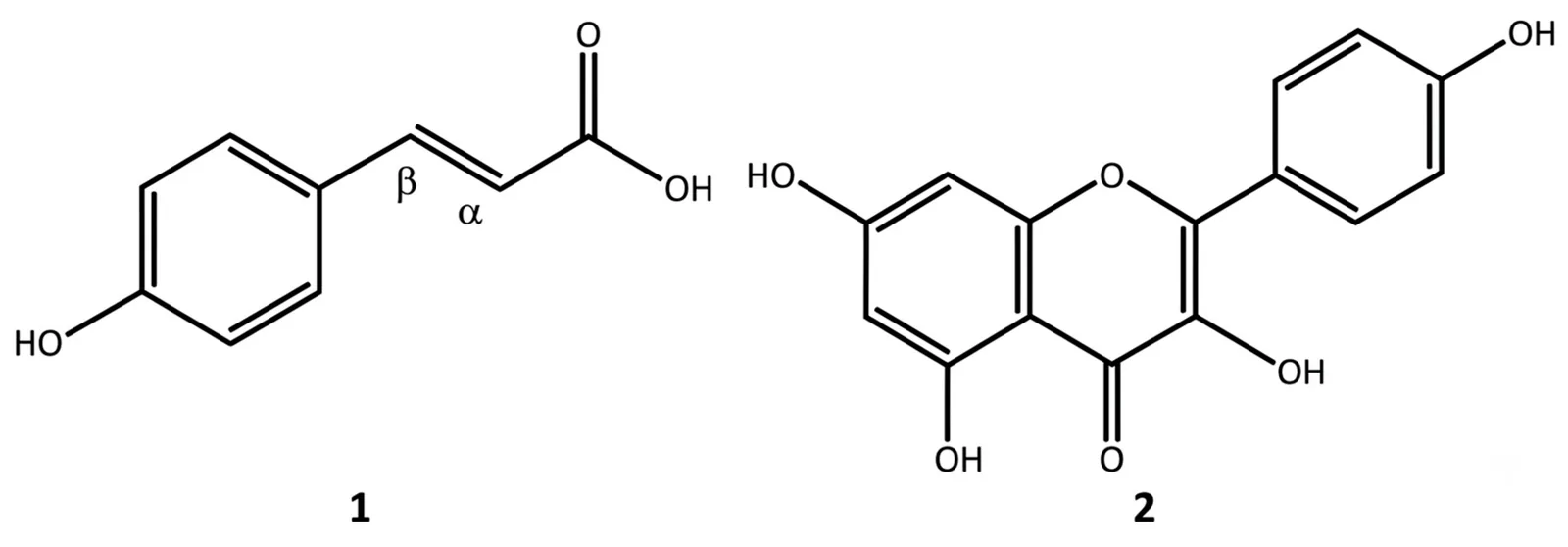
Chemical structures of compounds **1** and **2**.

Figure 1 shows the chromatogram of the MmAE extract, which exhibited a retention time of 10.84 min, consistent with those observed for fraction G4 (10.74 min) and compound **1** (10.05 min). The UV spectra associated with these peaks exhibited absorption maxima around 226 and 310 nm, characteristic of cinnamic acid, confirming the identity of this compound in the analyzed extract. The ^1^H NMR spectrum of p-coumaric acid exhibited two trans-coupled olefinic proton signals at δ 7.60 (d, *J* = 15.9 Hz, H-2′) and δ 6.27 (d, *J* = 15.9 Hz, H-1′), confirming the presence of a trans double bond. In addition, the aromatic region displayed the characteristic AA′BB′ spin system of a para-substituted phenyl ring, with signals at δ 7.44 (2H, d, *J* = 9.0 Hz, H-2/H-6) and δ 6.80 (2H, d, *J* = 8.6 Hz, H-3/H-5), supporting the identification of the compound as p-coumaric acid (Figure S6, Supplementary Materials). Negative-ion ESI-MS revealed a quasi-molecular ion at *m/z* 162.97 [M − H]^−^, consistent with the molecular formula C_9_H_7_O_3_ (Figure 2 and Figure 3).

- Identification of kaempferol (**2**)

**Figure 4:**
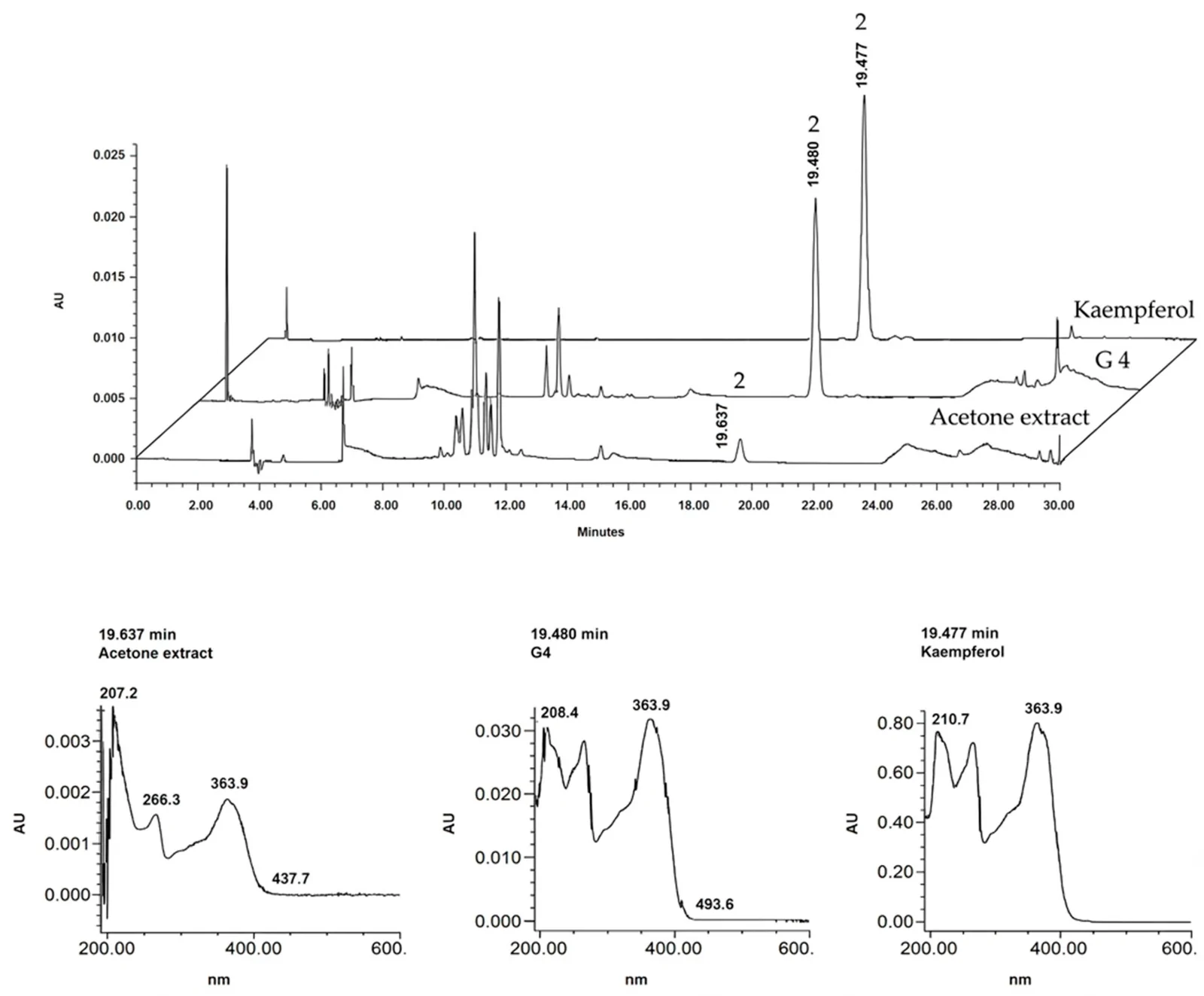
HPLC chromatograms (360 nm) of MmAE, G4, and kaempferol standard on a C18 column (acetonitrile/water gradient, 0.9 mL/min). The peak of kaempferol is indicated by (**2**). The UV spectrum (inset) shows maxima at 210, 266, and 363 nm. Rt: kaempferol = 19.47 min (standard), 19.48 min (G4), 19.63 min (MmAE).

Figure 4 shows the HPLC chromatogram of the extract, G4, and kaempferol. The MmAE extract had a retention time of 19.63 min, comparable to those obtained for fraction G4 (19.48 min) and compound **2** (19.44 min). The corresponding UV spectra showed absorption maxima characteristic of a flavonoid at 208, 266, and 363 nm, consistent with those reported for this flavonol, confirming its presence in the sample.

**Figure 5:**
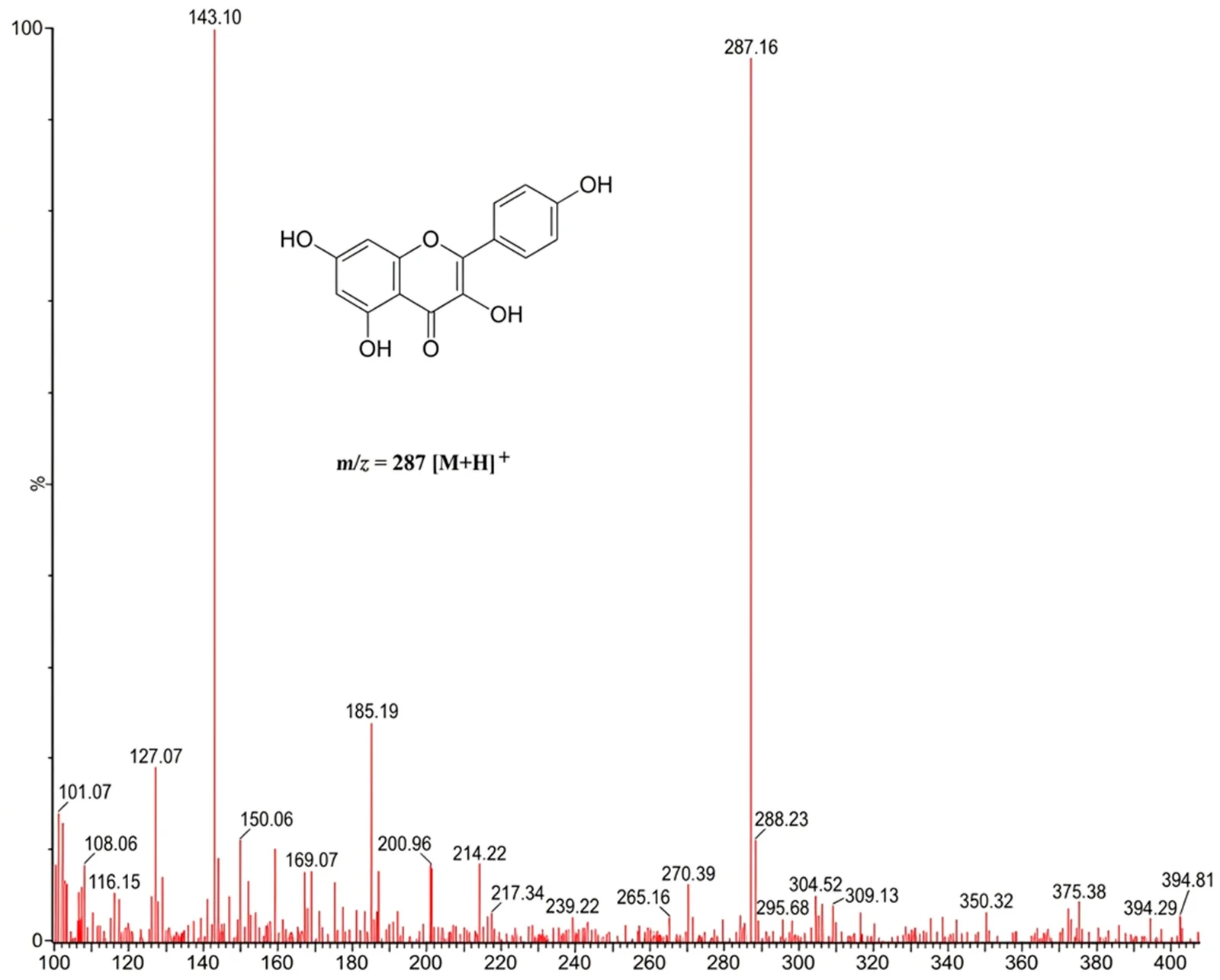
Mass spectrum of kaempferol (**2**).

The structure of compound **2** was characterized by using ^1^H and ^13^C nuclear magnetic resonance (NMR) spectroscopy, confirming its identity as kaempferol (15 mg, with 98.14% purity). The ^13^C NMR spectrum (125 MHz, CDCl_3_) showed 15 signals corresponding to its molecular structure, notably the signal of the conjugated carbonyl at δ 176.54 (C-4), as well as the characteristic resonances of oxygenated carbons at δ 163.90 (C-7), 162.10 (C-9), 160.12 (C-4′), and 157.80 (C-5). Likewise, aromatic signals were observed at δ 146.96 (C-2), 136.59 (C-3), 130.46 (C-2′/6′), 123.30 (C-1′), 116.28 (C-3′/5′), 104.10 (C-10), 99.08 (C-6), and 94.49 (C-8), consistent with the values reported for this flavonol [52,53,54]. ESI-MS of positive ions revealed quasi-molecular peaks at m/z 287 [M + H]^+^ for the molecular formula C_15_H_11_O_6_ (See Figure 3 and Figure 5). Additional spectroscopic data supporting the structural assignment of compounds **1** and **2** are provided in the Supplementary Materials (Figures S1–S6).

The structure of compound **2** was characterized by using ^1^H and ^13^C nuclear magnetic resonance (NMR) spectroscopy, confirming its identity as kaempferol (15 mg, with 98.14% purity). The ^13^C NMR spectrum (125 MHz, CDCl_3_) showed 15 signals corresponding to its molecular structure, notably the signal of the conjugated carbonyl at δ 176.54 (C-4), as well as the characteristic resonances of oxygenated carbons at δ 163.90 (C-7), 162.10 (C-9), 160.12 (C-4′), and 157.80 (C-5). Likewise, aromatic signals were observed at δ 146.96 (C-2), 136.59 (C-3), 130.46 (C-2′/6′), 123.30 (C-1′), 116.28 (C-3′/5′), 104.10 (C-10), 99.08 (C-6), and 94.49 (C-8), consistent with the values reported for this flavonol [52,53,54]. The ^1^H NMR spectrum (500 MHz, CDCl_3_) revealed an AA′BB′-type aromatic system corresponding to ring B, with signals at δ 8.15 (2H, d, *J* = 8.9 Hz, H-2′/6′) and δ 7.01 (2H, d, *J* = 8.9 Hz, H-3′/5′). In addition, two meta-coupled protons from ring A were observed at δ 6.53 (1H, d, *J* = 1.9 Hz, H-8) and δ 6.27 (1H, d, *J* = 2.0 Hz, H-6), as well as a characteristic signal of the chelated hydroxyl proton at δ 12.15 (1H, s, OH-5). Taken together, these spectroscopic data are consistent with the structure of kaempferol described in the literature [54,55,56]. The complete ^1^H and ^13^C NMR spectra of kaempferol are presented in the Supplementary Materials (Figures S1–S5). ESI-MS of positive ions revealed quasi-molecular peaks at *m/z* 287 [M + H]^+^ for the molecular formula C_15_H_11_O_6_ (See Figure 3 and Figure 5).

### 3.2. Calibration Curve LOD and LOQ

**Figure 6:**
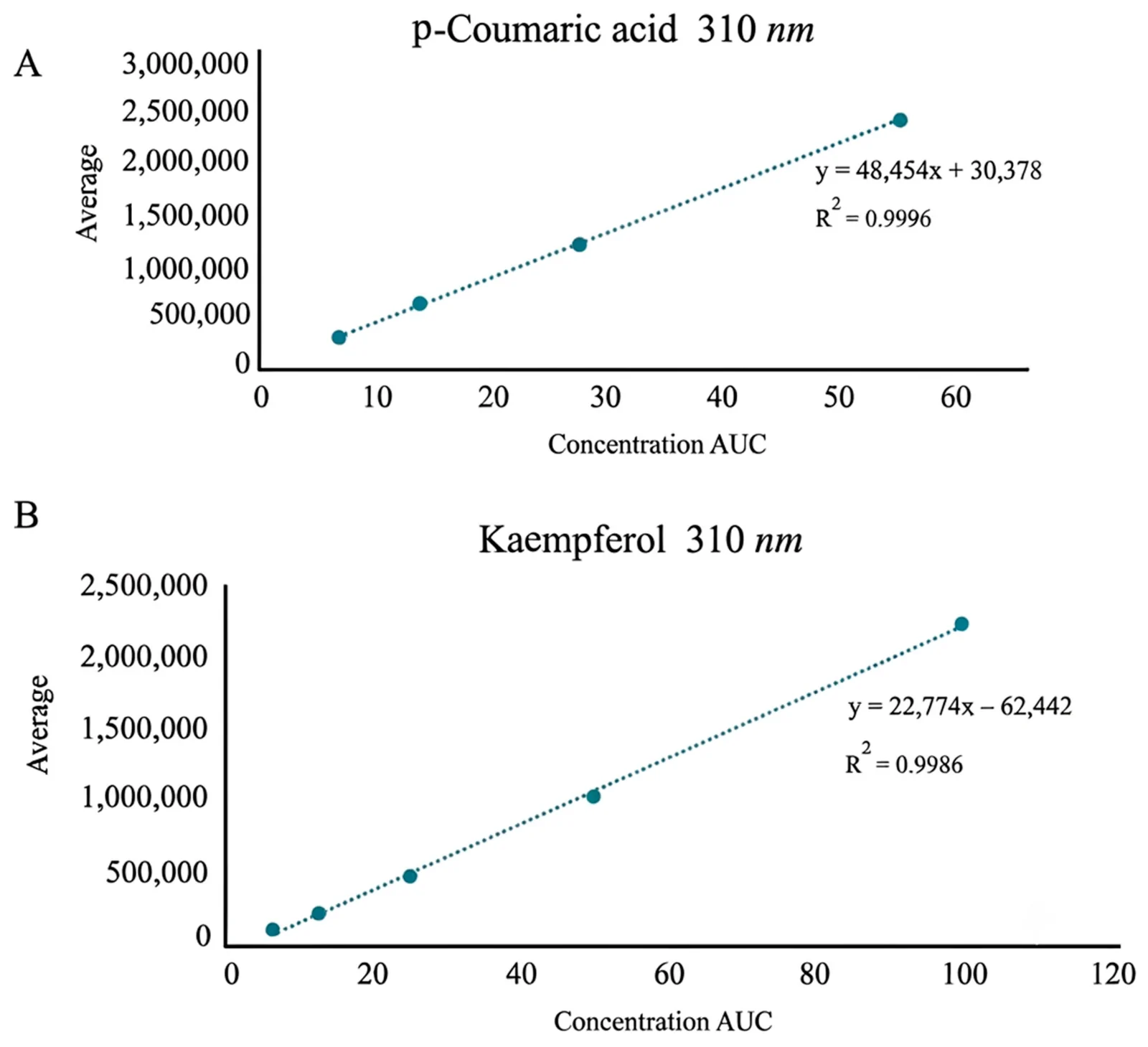
Calibration curves for (**A**) p-coumaric acid at 310 nm and (**B**) kaempferol at 360 nm. The relationship between concentration [mg/mL] and area under the curve (AUC) is shown. The data represents the average of three measurements (*n* = 3).

The curves were obtained by plotting the mean AUC against the concentration of each compound (Table 1). For p-coumaric acid, the relationship between concentration and AUC showed good linearity, with a linear regression equation of y = 48,454x + 30,378 and a correlation coefficient of R^2^ = 0.999. Similarly, kaempferol exhibited linear behavior, with a regression equation of y = 22,774x – 62,442 and a correlation coefficient of R^2^ = 0.998 (Figure 6). These results demonstrate high linearity across the evaluated concentration range, confirming the reliability and suitability of the analytical method for the quantification of both compounds. Quantification by HPLC-DAD revealed that G4 contained 3.13 mg of p-coumaric acid per g of fraction (0.313% *w*/*w*) and 19.45 mg of kaempferol per g of fraction (1.945% *w*/*w*). At the administered dose of 200 mg/kg, this corresponds to approximately 0.626 mg/kg of p-coumaric acid and 3.89 mg/kg of kaempferol. The LOD and LOQ results demonstrate high sensitivity and adequate linearity of the method for quantifying both compounds (Table 1).

### 3.3. Pharmacological Assays

#### 3.3.1. FST

**Figure 7:**
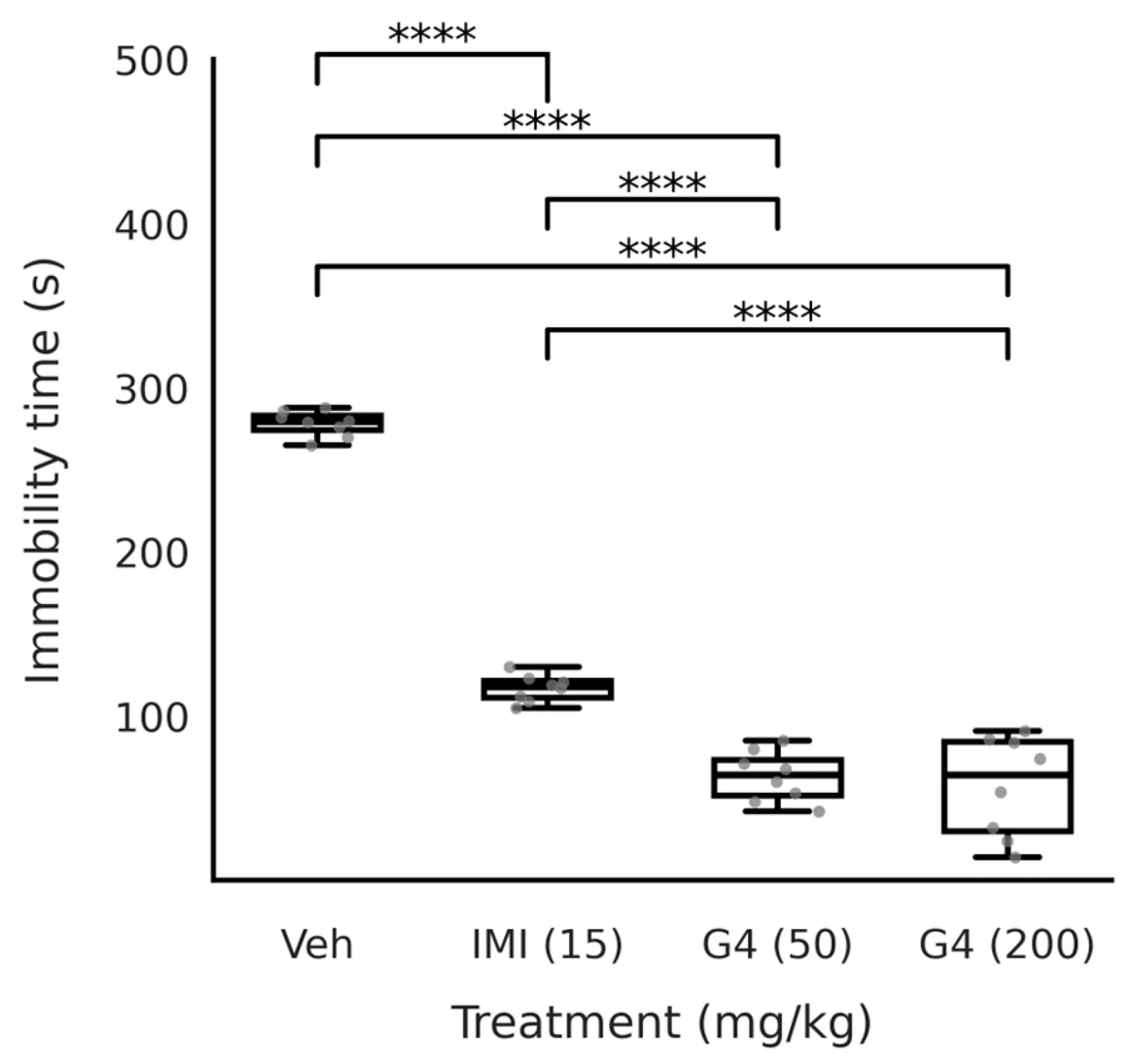
Effect of oral administration of fraction G4 from *M. mexicana* A. Juss. on the immobility time of CD-1 mice in the forced swimming test (FST). **** = *p* < 0.001 by one-way ANOVA followed by Tukey’s test (mean ± SD) with *n* = 8. IMI, imipramine hydrochloride; Veh, vehicle (100 µL/10 g); G4, Fraction 4 (50 and 200 mg/kg). Grey dots represent individual data points for each animal (*n* = 8 per group).

The immobility time differed significantly among the treatment groups. A one-way ANOVA revealed a main effect of treatment on immobility, F(3, 28) = 261.35, *p* < 0.001, with a very large effect size (η^2^p = 0.966). Figure 7 shows that the Veh group exhibited the highest levels of immobility (278.25 ± 7.76 s), while the IMI group (15 mg/kg) had a mean of 117.00 ± 8.09 s. This result is statistically significant compared to the Veh group (*p* < 0.001). G4 (50 mg/kg) also significantly reduced immobility time (63.38 ± 15.29 s) compared to the Veh group (*p* < 0.001). Similarly, G4 (200 mg/kg) significantly reduced immobility time (57.38 ± 30.67 s) compared to the Veh group (*p* < 0.001), with no statistically significant difference between the two doses (*p* > 0.05). Both doses of G4 produced significant reductions in immobility time compared to the vehicle control, with no statistically significant difference between the two doses (*p* > 0.05). The effects of both G4 doses differed significantly from the IMI group (*p* < 0.001).

#### 3.3.2. TST

**Figure 8:**
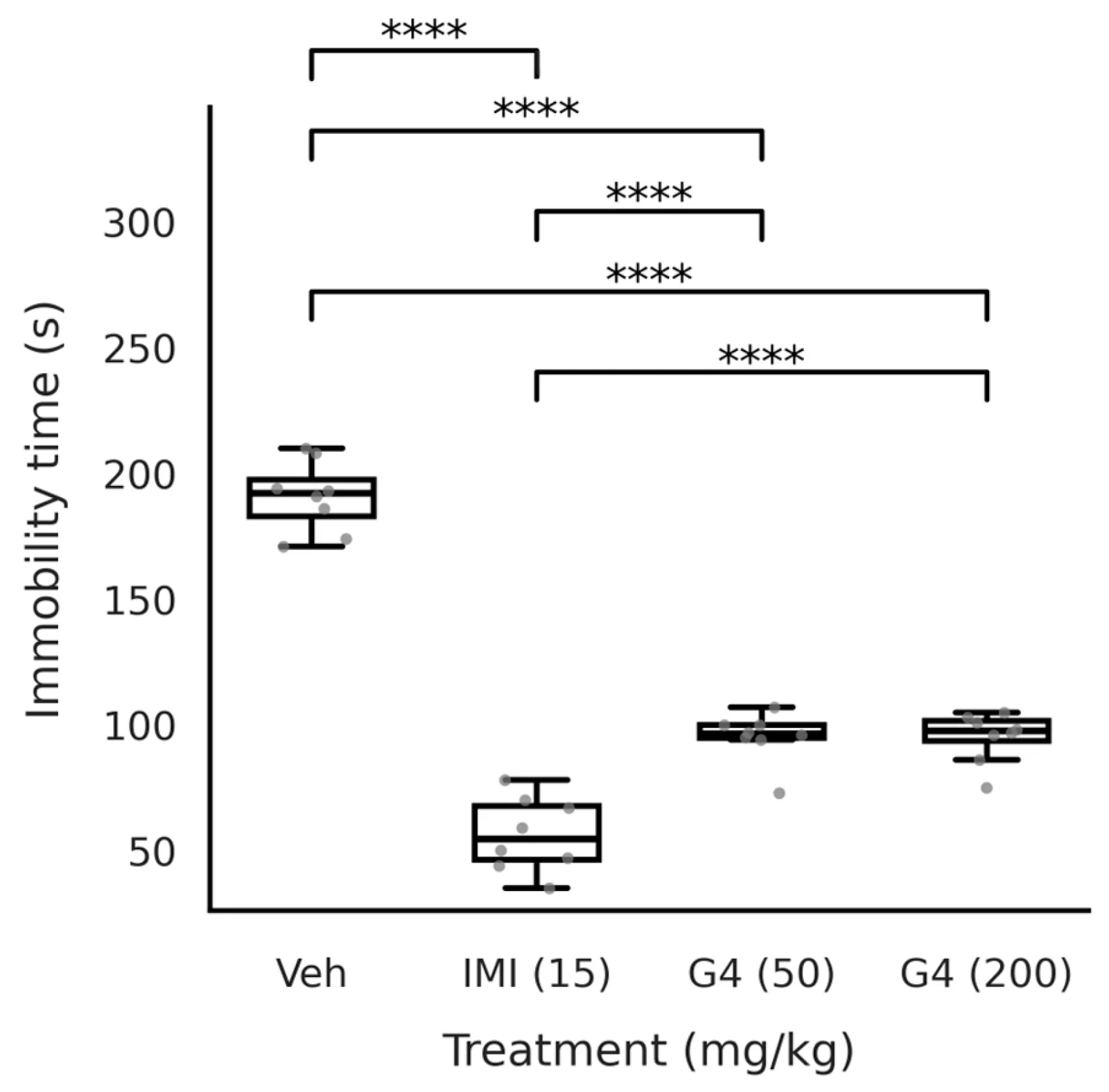
Effect of oral administration of fraction G4 from *M. mexicana* A. Juss. on the immobility time of CD-1 mice exposed to the tail suspension test (TST). **** = *p* < 0.001 by one-way ANOVA followed by Tukey’s test (mean ± SD) with *n* = 8. IMI, imipramine hydrochloride; Veh, vehicle (100 µL/10 g); G4, Fraction 4 (50 and 200 mg/kg). Grey dots represent individual data points for each animal (*n* = 8 per group).

In the tail-suspension test, the duration of immobility differed significantly among groups, showing a main effect of treatment in a one-way ANOVA (F(3, 28) = 172.67, *p* < 0.001), with a very large effect size (η^2^p = 0.949). Figure 8 shows that the Veh group exhibited the highest levels of immobility (190.88 ± 14.02 s), and the IMI group (15 mg/kg) had a mean of 56.25 ± 14.69 s. This result is statistically significant compared to the Veh group (*p* < 0.001), G4 (50 mg/kg) also significantly reduced immobility time (95.25 ± 09.88 s) compared to the Veh group (*p* < 0.001). Similarly, G4 (200 mg/kg) significantly reduced immobility time (95.12 ± 9.96 s) compared to the Veh group (*p* < 0.001), with no significant difference between the 50 and 200 mg/kg doses (*p* > 0.05). Both doses of G4 produced significant reductions in immobility time compared to the vehicle control, and their effects differed significantly from the IMI group (*p* < 0.001).

#### 3.3.3. OFT

**Figure 9:**
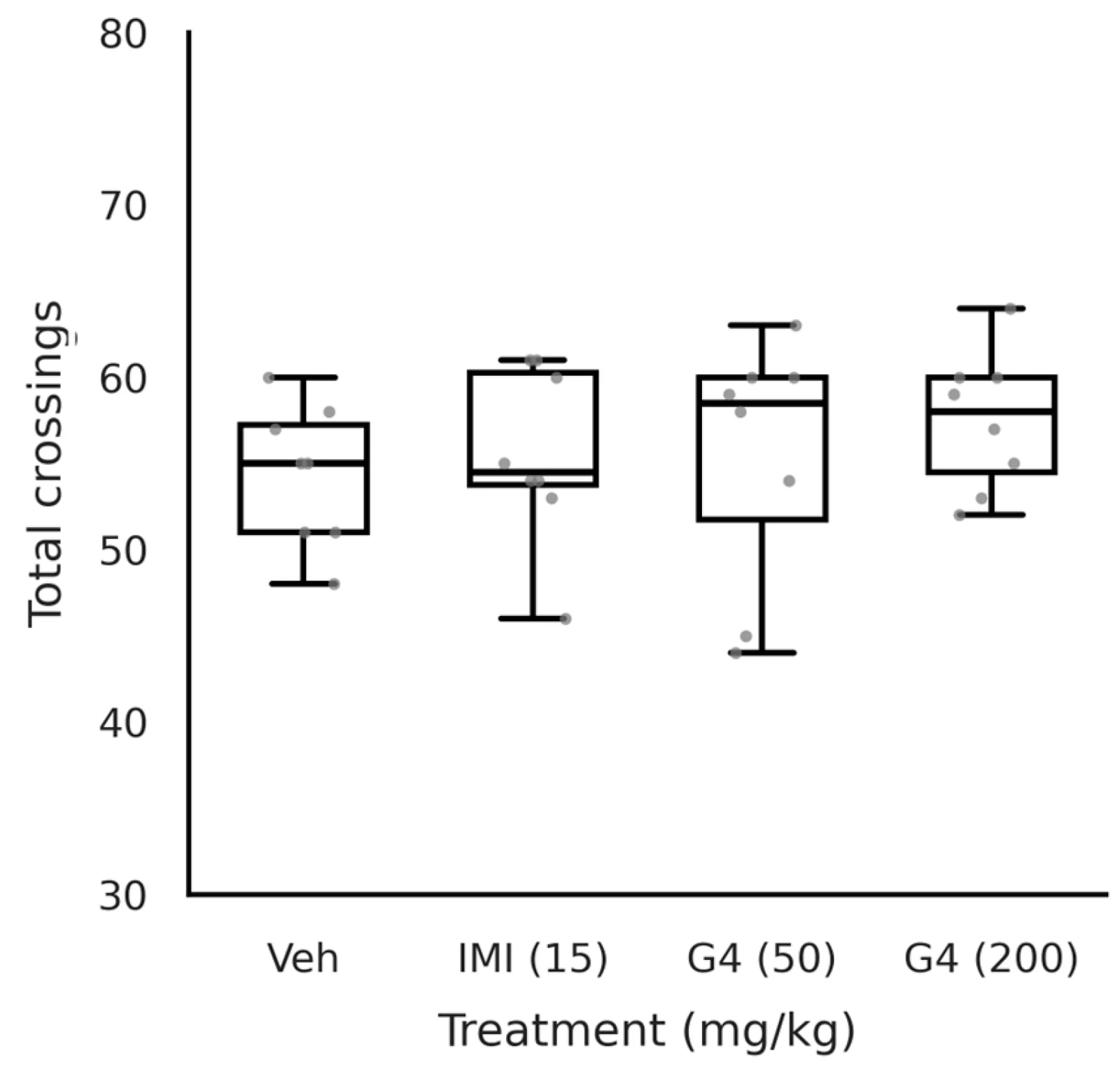
Effect of oral administration of fraction G4 on the total number of crossings in CD-1 mice exposed to OFT, compared with the Veh group. One-way ANOVA followed by Tukey’s test (mean ± SD) with *n* = 8. IMI, imipramine (15 mg/kg); Veh, vehicle (100 µL/10 g); and G4 (50 and 200 mg/kg). Grey dots represent individual data points for each animal (*n* = 8 per group).

The total number of crossings did not differ among the experimental groups. The one-way ANOVA did not show that the treatments altered locomotion (F(3, 28) = 0.497, *p* = 0.687; η^2^p = 0.051). Furthermore, Tukey’s post hoc analysis did not identify differences between pairs of groups (all comparisons *p* > 0.05). These data suggest that the treatments did not alter locomotor activity under the conditions evaluated (see Figure 9).

#### 3.3.4. Pentylenetetrazol-Induced Seizure Test (PTZt)

- Survival analysis

Following administration of pentylenetetrazol (PTZ, 100 mg/kg), the group that received the vehicle exhibited a rapid onset of seizures and death. The temporal course of the seizure response was markedly different in the groups that received diazepam (DZP, 1 mg/kg) and fraction G4 (50 and 200 mg/kg) compared to the vehicle group. In both the DZP and G4 treatments, the survival curves were shifted to the right, and fewer animals died during the observation period compared to the vehicle group (Table 2).

**Figure 10:**
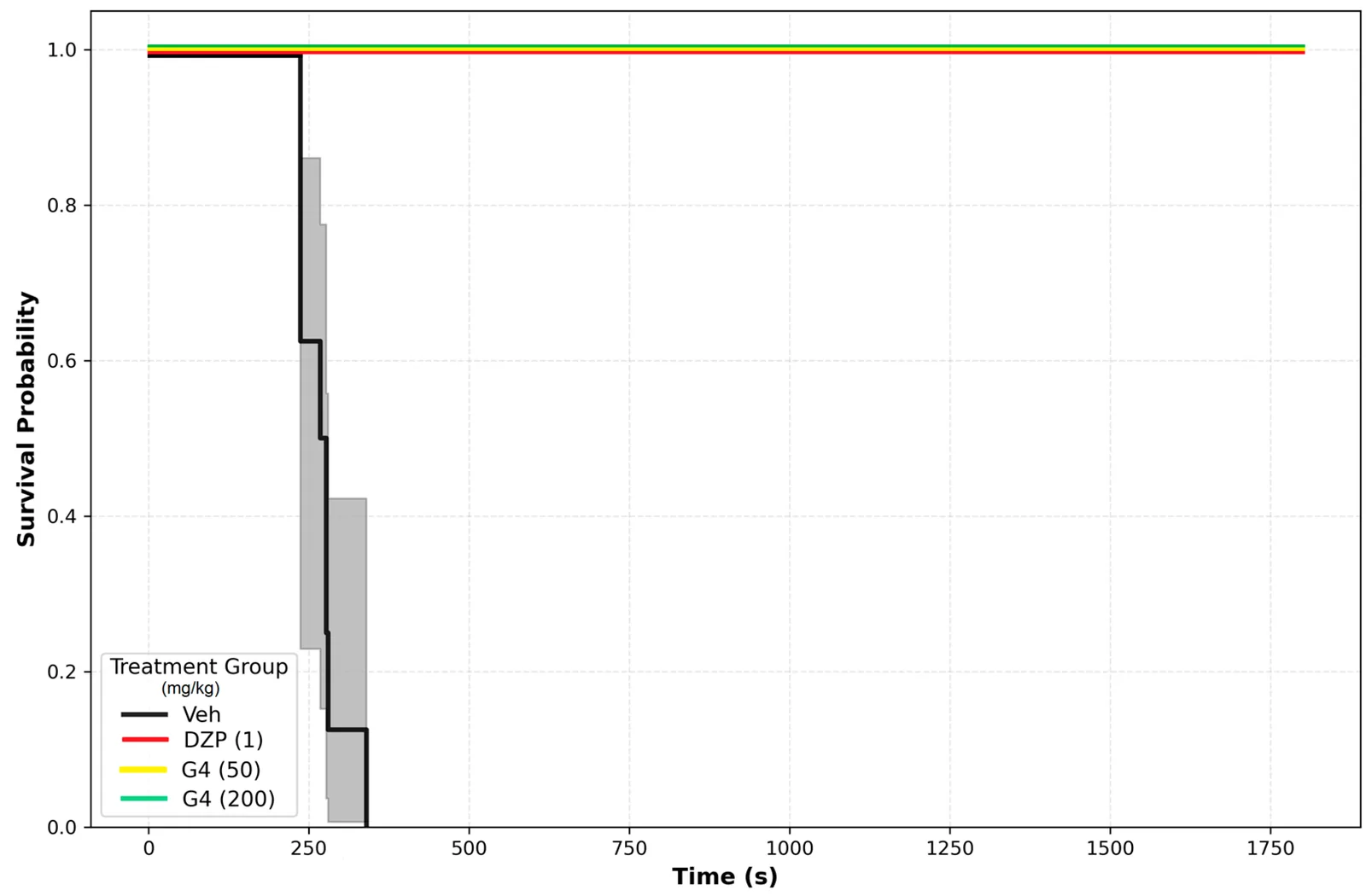
Kaplan–Meier survival curves in the pentylenetetrazol (PTZ)-induced acute seizure model. Animals were pretreated with vehicle (black bar), diazepam (DZP, 1 mg/kg; red bar), or extract G4 (50 mg/kg, yellow bar; 200 mg/kg, green bar) and subsequently challenged with PTZ. The vehicle group exhibited rapid seizure onset with a median survival time of 277 s. In contrast, all individuals from the treated groups survived the entire observation period, such that the median survival time could not be calculated. Shaded areas represent 95% confidence intervals. Log-rank test: χ^2^ = 17.0, *p* < 0.001 for all treatment groups vs. vehicle. *n* = 8 per group.

Survival curves (estimated using the Kaplan–Meier method and compared using the log-rank test with the Veh group as the negative control) showed that the DZP group had significantly higher survival compared to the Veh group (log-rank: χ^2^ = 17.0, *p* = 0.0001). Similarly, the G4 group (50 and 200 mg/kg) had significantly higher survival compared with the Veh group (for both doses: log-rank: χ^2^ = 17.0, *p* < 0.0001 vs. Veh). In the DZP and G4 groups, the median survival could not be estimated because less than 50% of the animals died under the experimental conditions (and, therefore, the median was not reached during the observation period). In contrast, in the Veh group, all animals died within the evaluation period, allowing for the calculation of a median survival of 277 s. These findings are consistent with a strong protective effect of DZP and G4 against PTZ-induced seizures and death. Figure 10 shows an overall comparison of the survival curves and indicates that the treatments evaluated (DZP 1 mg/kg, G4 50 mg/kg, and G4 200 mg/kg) differ significantly from the Veh group.

- Number of seizures

Table 3 shows the results of the analysis of the number of seizures using a one-way ANOVA followed by a Tukey post hoc test. For total seizures, there was a significant treatment effect (F = 50.516, *p* < 0.0001; η^2^p = 0.844). The groups treated with G4 (50 mg/kg) and G4 (200 mg/kg) showed a significant reduction compared to the Veh group (*p* < 0.05), with no differences between the two doses (*p* > 0.05), while DZP (1 mg/kg) showed a greater reduction (*p* < 0.0001). For tonic seizures, a treatment effect was also detected (F = 42.111, *p* < 0.0001; η^2^p = 0.819), with a similar pattern: the groups treated with G4 (50 mg/kg) and G4 (200 mg/kg) showed a significant reduction in tonic seizures compared to the Veh group (*p* < 0.05), with no difference between the two doses (*p* > 0.05), while DZP (1 mg/kg) showed a greater reduction in seizures (*p* < 0.0001). For the clonic seizure’s parameter, there was a significant overall effect (F = 3.785, *p* = 0.021; η^2^p = 0.289); DZP (1 mg/kg) significantly reduced (*p* < 0.05) the number of seizures compared to Veh and G4 (50 and 200 mg/kg), and there was no significant difference between the two doses of G4 (*p* > 0.05).

- Latency of seizures

Table 3 also shows a significant treatment effect (F = 70.26, *p* = 0.0001; η^2^ = 0.886) for the latency of onset of the first tonic seizure. The DZP group (1 mg/kg) (119.25 ± 10.10 s) had a longer latency time compared to the Veh group (*p* < 0.05). G4 50 mg/kg (106.38 ± 7.05 s) and G4 200 mg/kg (121.88 ± 10.71 s) significantly increased the latency of tonic seizures compared to the Veh group (*p* < 0.05). A statistically significant difference was observed between the two doses (*p* < 0.05), with the 200 mg/kg dose producing a longer latency that was comparable to that of DZP 1 mg/kg (*p* > 0.05). There was also a significant effect of treatment on latency of clonic seizures (F = 2456.07; *p* = 0.0001; η^2^ = 0.996). The DZP group (1 mg/kg) had significantly higher latency compared to the Veh group (88.75 ± 13.54 s) and both doses of G4 (*p* < 0.05). G4 50 mg/kg (110.00 ± 9.09 s) and G4 200 mg/kg (126.88 ± 11.67 s) significantly prolonged the latency of clonic seizures compared to Veh group (*p* < 0.05). The 200 mg/kg dose produced a significantly longer latency than the 50 mg/kg dose (*p* < 0.05), although both doses were significantly lower than that of the DZP group (*p* < 0.05).

## 4. Discussion

The present study demonstrates that a phenolic-enriched fraction (G4) obtained from *Malpighia mexicana* exhibits both antidepressant-like and anticonvulsant effects in murine models, without inducing alterations in spontaneous locomotor activity. These findings are consistent with the traditional use of species in Mexican ethnomedicine for the management of central nervous system disorders. They extend previous reports by providing evidence at the level of a chemically enriched fraction rather than crude extracts. Importantly, the dual neuropharmacological profile observed here suggests that G4 may target convergent pathways involved in mood regulation and seizure susceptibility.

The selection of G4 for detailed study was based on preliminary screening of all fractions (G1–G6) obtained from the acetone extract, in which G4 consistently showed superior neuropharmacological activity compared to the other fractions [20].

The reduction in immobility time observed in both the forced swimming test (FST) and tail suspension test (TST) indicates a robust antidepressant-like effect. Although both assays are widely used predictive tools in the screening of antidepressant drugs, there are methodological and physiological differences between them that may reflect partially distinct mechanisms of action [57,58]. Since locomotor activity was not significantly altered in the open field test (OFT), the behavioral changes are unlikely to be attributed to nonspecific psychostimulant effects. This distinction is critical, as increased locomotion can confound the interpretation of antidepressant-like activity. The magnitude of the effect observed for G4 was comparable to that of the reference drug, supporting its pharmacological relevance in these preclinical paradigms.

An important observation in this study is the lack of a clear dose–response relationship in several behavioral outcomes. In both the FST and TST, the effects of 50 mg/kg and 200 mg/kg were remarkably similar, with the higher dose not producing a significantly stronger reduction in immobility time compared to the lower dose. This pattern could be explained by several factors. First, it is possible that the 50 mg/kg dose already achieves near-maximal efficacy (i.e., a plateau effect), such that increasing the dose to 200 mg/kg does not result in further behavioral improvement. Second, some phytochemicals and natural product extracts exhibit biphasic (inverted U-shaped) responses, where moderate doses produce optimal effects and higher doses may be less effective or even counterproductive due to hormetic effects or receptor desensitization [59]. Third, dose-dependent changes in pharmacokinetics—such as saturable absorption, altered metabolism, or differential tissue distribution of key compounds—could contribute to non-linear pharmacological responses [58]. Finally, since G4 is a complex mixture of multiple phenolic compounds, dose-dependent changes in the relative bioavailability or interactions among constituents could modulate the overall pharmacological response. The absence of a clear dose–response relationship, together with the relatively small number of dose levels tested (only two), represents a limitation of the present study. Future investigations should include additional dose levels (e.g., 12.5, 25, 100 mg/kg) to fully characterize the dose–response profile and to identify the minimum effective dose and the dose range associated with maximal efficacy.

In addition to its antidepressant-like activity, G4 significantly delayed the onset of clonic and tonic seizures and reduced seizure severity in the PTZ-induced seizure model. Given that PTZ is widely used as a model of generalized seizures associated with GABAergic dysfunction, these findings suggest that G4 may modulate inhibitory neurotransmission or related pathways [51,52,53]. Our results suggest that G4 may indirectly modulate the excitatory/inhibitory balance in the central nervous system. A particularly relevant finding was the complete protection against mortality observed at the 200 mg/kg dose. This effect is comparable to that of diazepam in preventing lethal cardiorespiratory collapse, which is the leading cause of death in this model.

It is important to emphasize that the mechanistic interpretations presented below are hypothetical and are based on the known bioactivities of kaempferol and p-coumaric acid reported in the literature, as well as on the behavioral and pharmacological outcomes observed in this study. Direct experimental evidence for these mechanisms was not obtained in the present work, and therefore, these interpretations should be considered as working hypotheses that require validation through future biochemical, molecular, and neurochemical studies.

It is likely that the neuropharmacological effects observed for G4 are mediated by multiple interrelated mechanisms, consistent with the known bioactivities of its main constituents, kaempferol and p-coumaric acid. The structural identification of the major phenolic constituents supporting these interpretations is documented in the Supplementary Materials (Figures S1–S6). These mechanisms include antioxidant defense, modulation of monoaminergic neurotransmission, regulation of the GABAergic system, and anti-inflammatory pathways.

Antioxidant and Neuroprotective Mechanisms: Based on literature evidence, kaempferol is a potent antioxidant that reduces oxidative stress by scavenging reactive oxygen species (ROS) and upregulating endogenous antioxidant enzymes, such as superoxide dismutase (SOD), catalase (CAT), and glutathione peroxidase (GPx) [26]. Similarly, p-coumaric acid has been reported to protect neuronal cells from oxidative damage by inhibiting lipid peroxidation and improving mitochondrial function [27]. In the context of depression and epilepsy, oxidative stress plays a key role in neuronal damage and dysfunction [60]. Therefore, it is plausible that the phenolic compounds present in G4 could exert their antidepressant and anticonvulsant effects, at least in part, by reducing oxidative stress in key brain regions involved in the regulation of mood and susceptibility to epileptic seizures, such as the hippocampus and the prefrontal cortex. However, this hypothesis requires experimental confirmation through direct measurements of oxidative stress markers in brain tissue from G4-treated animals.

Modulation of monoaminergic systems: It has been reported that flavonoids, including kaempferol, can modulate monoaminergic neurotransmission by inhibiting monoamine oxidase (MAO) activity and increasing the synaptic availability of serotonin, norepinephrine, and dopamine [24]. This mechanism is consistent with the antidepressant-like effects observed in the FST and TSTs, which are behavioral paradigms sensitive to changes in serotonergic and noradrenergic transmission [57]. The reduction in immobility time induced by G4, comparable to that of imipramine (a tricyclic antidepressant that inhibits the reuptake of serotonin and norepinephrine), suggests the possibility that G4 may similarly enhance monoaminergic signaling, potentially through MAO inhibition or modulation of monoamine transporters. However, direct evidence for this mechanism—such as measurements of monoamine levels, MAO activity, or transporter function in brain tissue—was not obtained in the present study and should be addressed in future investigations.

Anti-inflammatory and neuroimmunomodulatory effects: Chronic neuroinflammation is a common pathophysiological feature of both depression and epilepsy. Proinflammatory cytokines, such as TNF-α, IL-1β, and IL-6, contribute to neuronal hyperexcitability, synaptic dysfunction, and alterations in neurotransmitter metabolism [60]. Based on literature evidence, p-coumaric acid has been shown to exert anti-inflammatory effects by inhibiting the AGE-RAGE signaling pathway and reducing the release of pro-inflammatory mediators [28]. Furthermore, both kaempferol and p-coumaric acid have been reported to modulate the TLR4/NF-κB pathway, reducing neuroinflammation and promoting neuroprotection [29]. Consequently, it is plausible that the dual antidepressant and anticonvulsant effects of G4 could be partly attributed to its anti-inflammatory properties, potentially restoring the neuroimmunological balance in the central nervous system. This hypothesis, however, requires experimental validation through measurements of cytokine levels and inflammatory markers in brain tissue from treated animals.

Synergistic interactions: It is important to consider that the observed pharmacological effects of G4 may not be attributable solely to kaempferol and p-coumaric acid individually but could also involve synergistic or additive interactions among the multiple phenolic components present in the fraction. This phenomenon is well documented in phytomedicine, where mixtures of bioactive compounds often exhibit greater efficacy compared to individual compounds due to complementary mechanisms of action, improved bioavailability, or multi-target effects [61]. The complete protection against mortality observed at 200 mg/kg, despite the relatively modest concentration of kaempferol in the fraction (19.45 mg/mL in the fraction, corresponding to approximately 3.9 mg/kg of kaempferol at the 200 mg/kg dose), is consistent with the hypothesis of pharmacodynamic synergy. However, this interpretation remains speculative in the absence of direct comparative studies with the isolated compounds. Future studies should systematically evaluate the individual and combined contributions of the major compounds to determine whether synergistic or additive effects are responsible for the observed neuropharmacological activity.

The absence of a clear dose–response relationship should also be interpreted considering the potential influence of pharmacokinetic factors, including saturable absorption, altered metabolism, and tissue distribution of bioactive constituents, which may contribute to non-linear pharmacological responses in complex natural product mixtures [62]. Because only two dose levels were evaluated, additional studies will be necessary to further characterize the pharmacological profile of G4.

Phytochemical analysis by HPLC-DAD revealed that G4 is enriched in phenolic compounds, particularly kaempferol and p-coumaric acid, both of which have been previously associated with neuroprotective and neuromodulatory properties in other species. Flavonoids such as kaempferol have been reported to exert antidepressant-like and anticonvulsant effects in experimental models, potentially through antioxidant, monoaminergic, and GABAergic mechanisms [24,25,26] effects that may contribute to the observed pharmacological activity [26]. The relative contribution of individual compounds and potential synergistic interactions within the fraction remain to be elucidated. Similarly, p-coumaric acid has demonstrated neuroprotective and antidepressant-like effects through anti-inflammatory and antioxidant pathways [27,28,29]. Based on this literature evidence, it is plausible that these compounds contribute to the pharmacological activity observed for G4. However, it is important to emphasize that since only the G4 fraction was pharmacologically evaluated and not the isolated compounds, the specific contribution of kaempferol and p-coumaric acid to the observed effects remains hypothetical. The relative contribution of individual compounds, as well as potential synergistic or additive interactions among the multiple phenolic constituents present in the fraction, remain to be elucidated through future studies with isolated compounds and defined mixtures. The dual activity observed in this study is particularly relevant from a translational perspective. Depression and epilepsy frequently co-occur and share overlapping neurobiological mechanisms, including alterations in neurotransmitter systems, neuroinflammation, and oxidative stress [29,55]. Therefore, compounds or fractions capable of modulating multiple pathways may offer therapeutic advantages over single-target agents. In this context, the activity of G4 supports the exploration of *M. mexicana* as a potential source of multi-target neuroactive compounds.

A key point for the technical discussion is that the efficacy of G4 (200 mg/kg) is comparable to doses of 10–20 mg/kg of pure kaempferol reported in the literature [26]. Although the dose of the fraction is numerically higher, this suggests functional enrichment or pharmacodynamic synergy. The fraction achieves total survival using a proportion of the flavonoid that, in isolation, might be insufficient. This reinforces the viability of using standardized fractions that improve bioavailability or modulate multiple targets simultaneously [61,63]. These results are consistent with studies in other species of the Malpighiaceae family, such as *Byrsonima crassifolia* and *Heteropterys brachiata*, whose phenolic-rich extracts have demonstrated antidepressant and anticonvulsant effects in animal models [21,22]. This validates the use of enriched fractions to improve chemical reproducibility.

Although kaempferol and p-coumaric acid were identified as the main components of fraction G4, the observed antidepressant and anticonvulsant effects cannot be attributed exclusively to the synergy of these two compounds; it is important to isolate each compound to determine its pharmacological activity and to observe whether each acts independently.

One limitation of the present study is that only two doses of G4 were evaluated. Although both doses produced significant neuropharmacological effects, this experimental design does not allow a complete characterization of the dose–response relationship. As discussed above, the similarity in efficacy between the 50 and 200 mg/kg doses in several behavioral outcomes suggests that the dose–response curve may have reached a plateau or may be biphasic. However, without additional dose levels, this interpretation remains speculative. Future studies should include additional dose levels (e.g., 12.5, 25, 100 mg/kg) to determine the minimum effective dose, maximal efficacy, shape of the dose–response curve, and pharmacological potency of the fraction.

Furthermore, although kaempferol and p-coumaric acid were identified as the main components of G4, and the literature supports their neuropharmacological potential, it would be important for future research to evaluate these compounds individually. Additionally, while we have proposed several mechanistic hypotheses based on the known bioactivities of kaempferol and p-coumaric acid and on the observed behavioral results, it would be important for future research to conduct direct biochemical, molecular, neurochemical, inflammatory, or oxidative stress experiments to confirm these mechanisms. Therefore, the mechanistic interpretations presented in the discussion should be considered working hypotheses that require experimental validation through specific mechanistic studies in future research.

The mechanistic hypotheses proposed here—involving antioxidant defense, monoaminergic modulation, anti-inflammatory pathways, and synergistic interactions—provide a solid foundation for future research. However, the present study has limitations, including the use of only two dose levels, which precludes a complete characterization of the dose–response relationship, and the lack of direct mechanistic experiments to confirm the proposed pathways. Nevertheless, the dual neuropharmacological profile of G4, combined with its favorable phytochemical composition, positions this phenolic-enriched fraction as a promising candidate for the development of phytopharmaceuticals targeting depression and epilepsy. Future investigations should focus on validating the proposed mechanisms and evaluating the translational potential of this fraction.

## 5. Conclusions

The G4 fraction of *M. Mexicana*, which is enriched in kaempferol and p-coumaric acid, exhibits antidepressant-like effects in the FST and TST models, comparable to those of the reference drug imipramine. It also demonstrates anticonvulsant effects against PTZ-induced seizures, with the 200 mg/kg dose providing complete protection against mortality in the acute seizure model. However, the similar efficacy observed at 50 and 200 mg/kg in several behavioral paradigms suggests that the dose–response relationship requires further characterization with additional dose levels to identify the optimal therapeutic window.

Based on literature evidence, kaempferol and p-coumaric acid are plausible candidates for mediating these effects; however, since only the G4 fraction was pharmacologically evaluated and not the isolated compounds, direct causality has not been established. These findings support the traditional use of guachocote for nervous disorders and suggest that G4 is a promising candidate in the search for and development of phytopharmaceuticals against depression and epilepsy, warranting further mechanistic and compound-specific investigations.

To conclusively address the mechanistic questions raised by our findings and to advance the translational potential of G4, future studies should focus on: (1) evaluating the effects of G4 on neurotransmitter levels (serotonin, dopamine, norepinephrine) and their metabolites in specific brain regions (hippocampus, prefrontal cortex, striatum) using HPLC-ECD or microdialysis techniques, which would directly test the monoaminergic modulation hypothesis; (2) assessing GABA[A] receptor binding affinity and chloride influx in neuronal cultures to confirm GABAergic modulation, thereby validating the proposed mechanism underlying the anticonvulsant effects; (3) measuring markers of oxidative stress (malondialdehyde, reduced glutathione, SOD, CAT) and neuroinflammation (TNF-α, IL-1β, IL-6, NF-κB) in brain tissue from treated animals to evaluate the antioxidant and anti-inflammatory hypotheses; (4) investigating the MAO-A and MAO-B inhibitory activity of individual compounds and the G4 fraction using in vitro enzymatic assays to determine whether monoamine oxidase inhibition contributes to the antidepressant-like effects; (5) performing transcriptomic or proteomic analyses to identify signaling pathways (e.g., PKA/CREB/BDNF, AGE-RAGE, TLR4/NF-κB) modulated by G4 treatment, which would provide unbiased mechanistic insights; and (6) conducting pharmacokinetic studies to determine the bioavailability and brain penetration of kaempferol and p-coumaric acid following oral administration of the fraction, which is essential for assessing its therapeutic viability. Collectively, these studies will elucidate the precise molecular targets and pathways involved in the neuropharmacological effects of G4 and will establish a solid foundation for its potential clinical translation.

## Figures and Tables

**Table 1:** Areas under the curve (AUC) and concentrations of compound **1** at 310 nm and compound **2** at 360 nm in MmAE and G4.

Compound	Sample	Concentration [mg/g]
p-coumaric acid	MmAE	1.36
p-coumaric acid	G4	3.13
Kaempferol	MmAE	3.68
Kaempferol	G4	19.45

**Table 2:** Summary of Kaplan–Meier survival analysis and log-rank test results comparing treatment groups to vehicle control in the pentylenetetrazol-induced seizure model.

Treatment Group	*n*	Median Survival (s)	95% CI	Log-Rank (χ^2^)	*p*-Value
Veh 100 µL/10 g	8	277	237–340	—	—
DZP 1 mg/kg	8	NR	NR	17.0	<0.001
G4 50 mg/kg	8	NR	NR	17.0	<0.001
G4 200 mg/kg	8	NR	NR	17.0	<0.001

Abbreviations: Veh, vehicle; DZP, diazepam; G4, fraction G4; NR, not reached; CI, confidence interval. All comparisons are vs. vehicle control using the log-rank test. *p* < 0.001 indicates statistical significance at the α = 0.05 level. —, not calculated (vehicle group served as the reference control for the log-rank test).

**Table 3:** Effects on the number and latency of seizures in the pentilentetrazol-induced seizure model.

Treatment Group	Number of Tonic Seizures	Number of Clonic Seizures	Total Number of Seizures	Latency to First Tonic Seizure (s)	Latency to First Clonic Seizures (s)
Veh 100 µL/10 g	67.38 ± 05.50 ^a^	04.38 ± 01.41 ^a^	72.75 ± 04.86 ^a^	63.63 ± 04.57 ^a^	88.75 ± 13.54 ^a^
DZP 1 mg/kg	25.13 ± 08.27 ^b^	01.88 ± 01.13 ^b^	25.63 ± 10.03 ^b^	119.25 ± 10.10 ^b^	495.25 ± 09.39 ^b^
G4 50 mg/kg	42.50 ± 10.10 ^c^	03.63 ± 02.20 ^a^	46.38 ± 09.33 ^c^	106.38 ± 07.05 ^c^	110.00 ± 09.09 ^c^
G4 200 mg/kg	49.38 ± 05.60 ^c^	02.88 ± 01.25 ^a^	52.25 ± 05.18 ^c^	121.88 ± 10.71 ^b^	126.88 ± 11.67 ^d^

The results are presented as mean ± SD. Different superscript letters within the same column indicate statistically significant differences among treatments (one-way ANOVA followed by Tukey’s post hoc test, α = 0.05). Veh: vehicle; DZP: diazepam; G4: fraction G4.

## Data Availability

The data are available upon reasonable request.

## Supplementary Materials

The following supporting information can be downloaded at: https://www.mdpi.com/article/10.3390/neurosci7040088/s1, Figure S1: TLC and HPLC analysis of the acetone extract of *Malpighia mexicana* recorded at 310 nm, p-coumaric acid (**1**), kaempferol (**2**); Figure S2: TLC and HPLC analysis of group 4 recorded at 310 nm, p-Coumaric acid (**1**), Kaempferol (**2**); Figure S3: TLC and HPLC analysis of fractions F3 and F5, observed at 310 nm, showing the presence of the phenolic compound F3 (A) p-Coumaric acid and F5 (C) Kaempferol, as well as the commercial standards observed at 310 nm (B) p-Coumaric acid (**1**) and (D) Kaempferol (**2**); Figure S4: ^1^H NMR (Acetone d6, 500 MHz) of Kaempferol (**2**); Figure S5: ^13^C NMR (Acetone d6, 125 MHz) of Kaempferol (**2**); Figure S6. ^1^H NMR (CD_3_OD, 80 MHz) of p-Coumaric acid (**1**).

## Abbreviations

The following abbreviations are used in this manuscript:

AGE-RAGE	Advanced Glycation End-products-Receptor for Advanced Glycation End-products
ANOVA	Analysis of Variance
AUC	Area Under the Curve
BDNF	Brain-Derived Neurotrophic Factor
CDCl_3_	Deuterated Chloroform (used as a solvent in NMR)
CI	Confidence Interval
CREB	cAMP Response Element-Binding Protein
DZP	Diazepam
ESI-MS	Electrospray Ionization Mass Spectrometry
FST	Forced Swimming Test
G1 to G6	Groups of fractions obtained by chromatography (G4 is the study fraction)
HPLC	High Performance Liquid Chromatography
HPLC-DAD	High Performance Liquid Chromatography-Diode Array Detector
HUMO	Herbarium of the University of Morelos
IMI	Imipramine
i.p.	Intraperitoneal
m.a.s.l.	Meters above sea level
MmAE	*Malpighia mexicana* Acetone Extract
NMR	Nuclear Magnetic Resonance
NOM	Official Mexican Standard (Norma Oficial Mexicana)
NR	Not Reached (referring to median survival)
OFT	Open Field Test
PKA	Protein Kinase A
p.o.	Per os (by mouth/oral)
PTZ	Pentylenetetrazol
PTZt	Pentylenetetrazol-induced Seizure Test
R^2^	Coefficient of determination
TLR	Toll-Like Receptors
TLC	Thin-Layer Chromatography
TST	Tail Suspension Test
UAEM	Autonomous University of the State of Morelos
UPLC-MS	Ultra Performance Liquid Chromatography-Mass Spectrometry
UV	Ultraviolet
Veh	Vehicle
WHO	World Health Organization
η^2^p	Partial Eta Squared (measure of effect size)
