# Supplementary material for: Antidepressant-like and Anticonvulsant Effects of a Phenolic-Enriched Fraction from Malpighia mexicana in Murine Models
Source: NeuroSci. 2026 Aug 10;7(4):88. doi: 10.3390/neurosci7040088 (PMC13516562; doi:10.3390/neurosci7040088)
Supplement: Supplementary file 1 [file neurosci-07-00088-s001.zip › neurosci-4450149-supplementary.pdf]

Supplementary data

Article

Antidepressant-like and Anticonvulsant Effects of a Phenolic-Enriched Fraction from *Malpighia mexicana* in Murine Models

David Osvaldo Salinas-Sánchez [1,2], Manasés González-Cortazar [3,*], Franceli Itzel Batalla-Martinez [4,5], Alejandro Zamilpa [3], Maura Téllez-Téllez [5], María Guadalupe Valladares-Cisneros [6], César Sotelo-Leyva [7], Rodolfo Figueroa-Brito [8], Ma Dolores Pérez-García [3] and Dante Avilés-Montes [9,*]

Figures

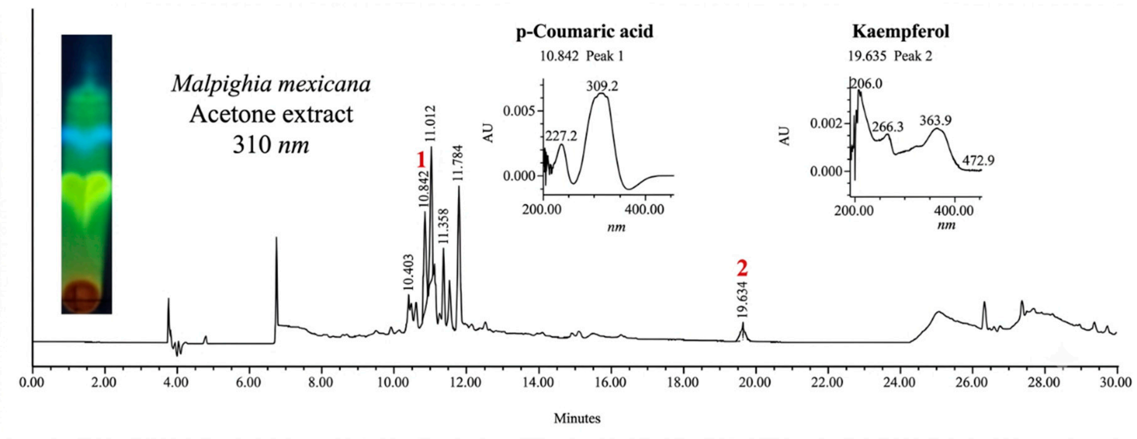

Figure S1. TLC and HPLC analysis of the acetone extract of *Malpighia mexicana* recorded at 310 *nm*, p-coumaric acid (1), kaempferol (2).

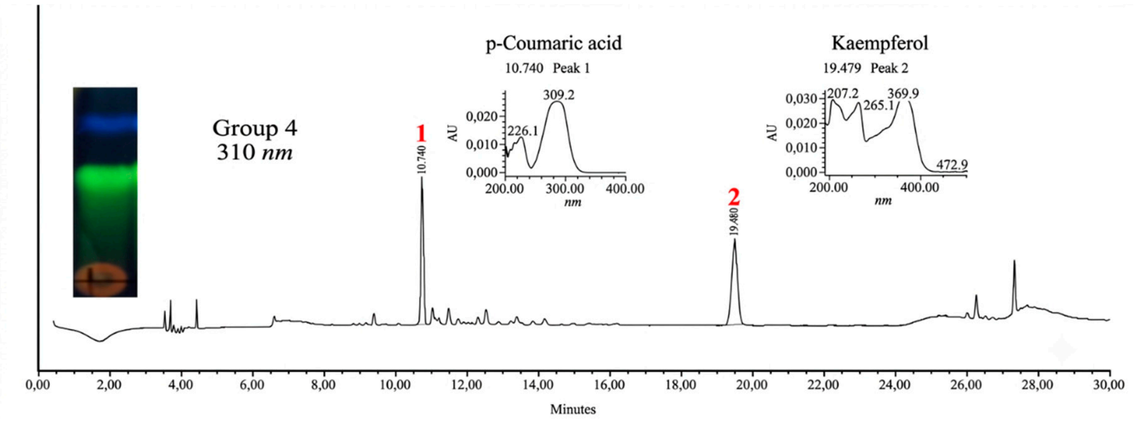

Figure S2. TLC and HPLC analysis of group 4 recorded at 310 *nm*, p-Coumaric acid (1), Kaempferol (2).

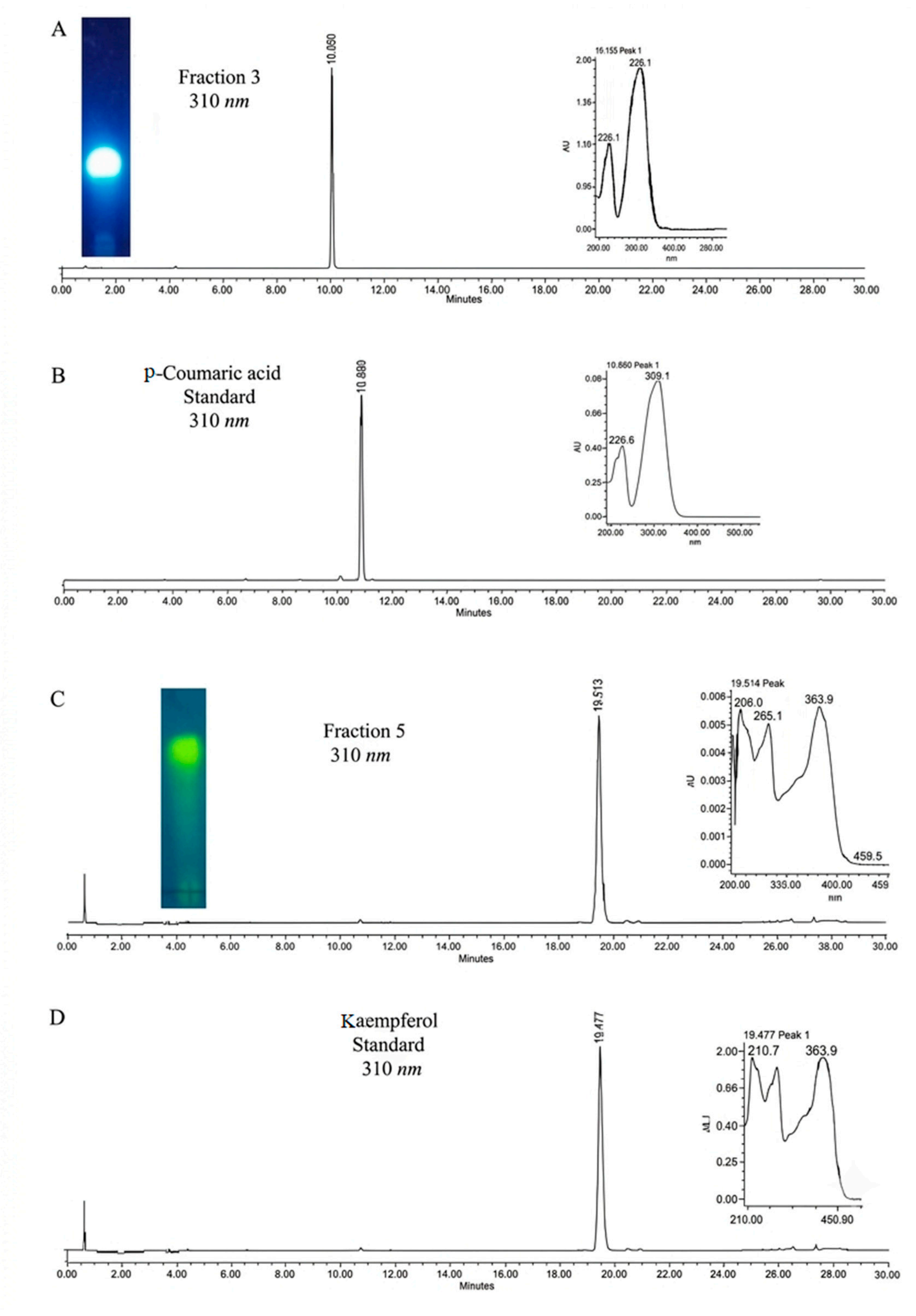

Figure S3. TLC and HPLC analysis of fractions F3 and F5, observed at 310 *nm*, showing the presence of the phenolic compound F3 (A) p-Coumaric acid and F5 (C) Kaempferol, as well as the commercial standards observed at 310 nm (B) p-Coumaric acid (**1**) and (D) Kaempferol (**2**).

Kaempferol NMR spectroscopic data

^{13}C NMR (Acetone d6, 125 MHz); δ 176.54 (C-4), 163.90 (C-7), 162.10 (C-9), 160.12 (C-4′), 157.80 (C-5), 146.96 (C-2), 136.59 (C-3), 130.46 (C-2′,6′), 123.30 (C-1′), 116.28 (C-3′,5′), 104.10 (C-10), 99.08 (C-6), 94.49 (C-8). ^{1}H NMR (Acetone d6, 500 MHz) δ 8.15 (2H, d, J = 8.9 Hz, H-2′,6′), 7.01 (2H, d, J = 8.9 Hz, H-3′,5′), 6.53 (1H, d, J = 1.9 Hz, H-8), 6.27 (1H, d, J = 2.0 Hz, H-6), 12.15 (1H, s, OH-5).

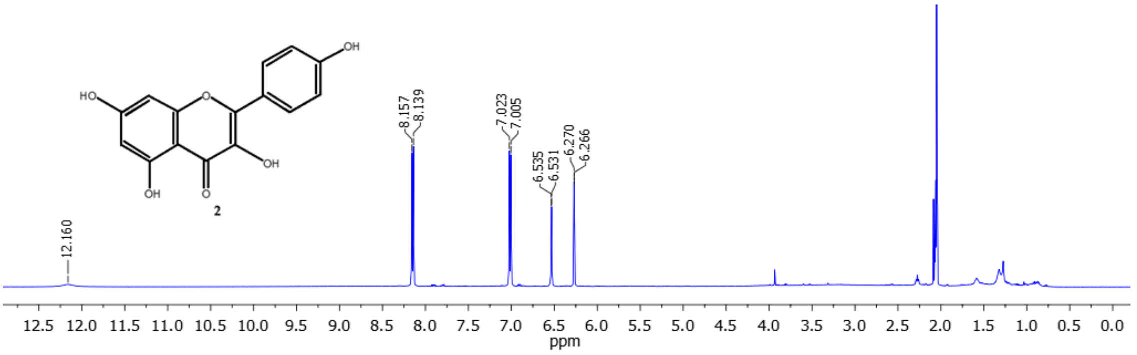

Figure S4. ^{1}H NMR (Acetone d6, 500 MHz) of Kaempferol (**2**).

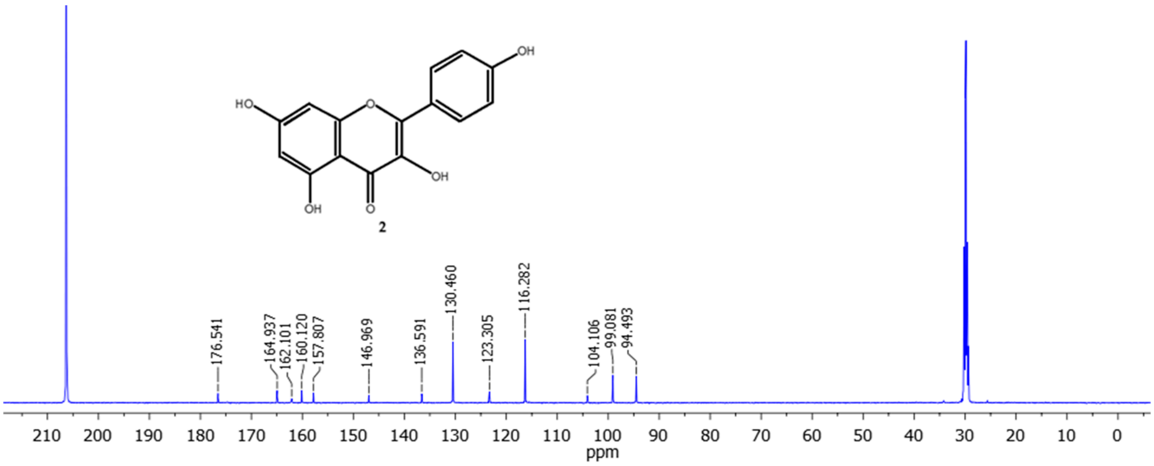

Figure S5. ^{13}C NMR (Acetone d6, 125 MHz) of kaempferol (**2**).

p-Coumaric acid NMR spectroscopy data

^{1}H NMR (CD_3OD, 80 MHz) δ 6.27(1H, d, J = 15.9 Hz, H-2′), 7.60 (1H, d, J = 16.9 Hz, H-1′), 7.44 (2H, d, J = 9 Hz, H-2, H-6), 6.80 (2H, d, J = 8.6 Hz, H-3, H-5).

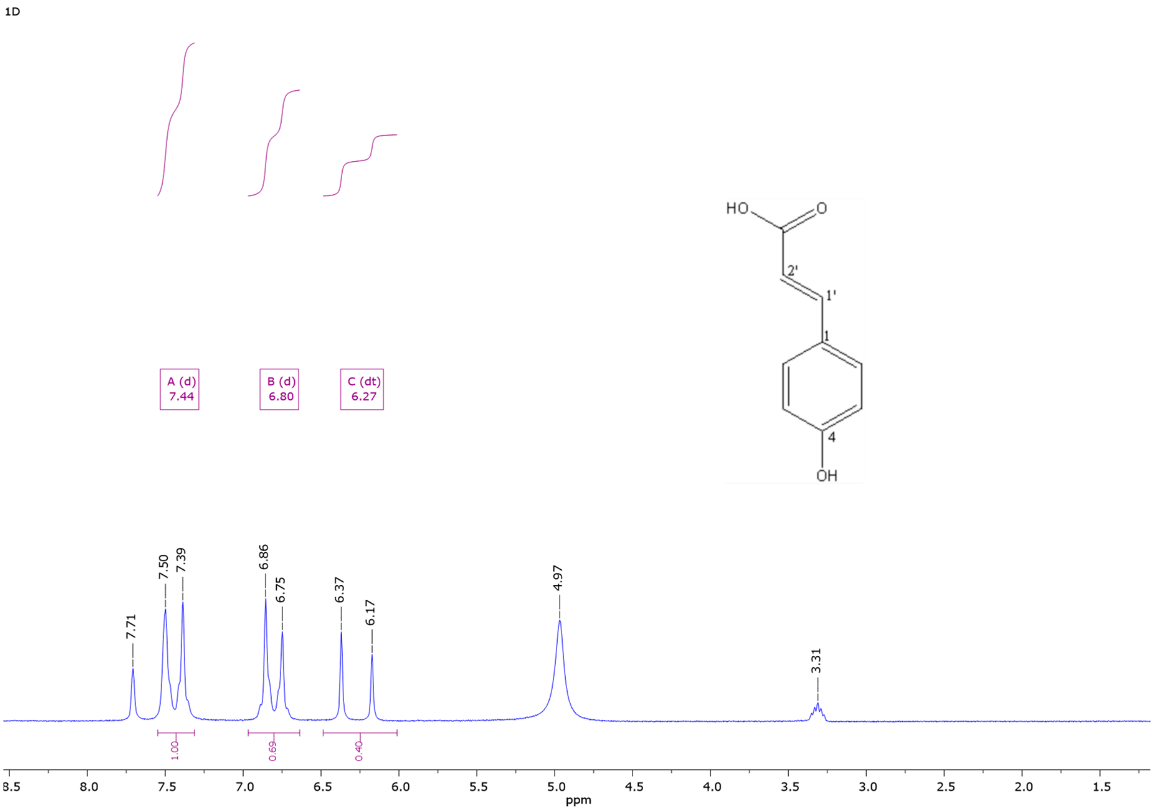

Figure S6. ^{1}H NMR (CD_3OD, 80 MHz) of p-Coumaric acid (**1**).
